# The response of regional well-being to place-based policy interventions

**DOI:** 10.1016/j.regsciurbeco.2022.103830

**Published:** 2022-11

**Authors:** Antonella Rita Ferrara, Lewis Dijkstra, Philip McCann, Rosanna Nisticó

**Affiliations:** a European Commission, Joint Research Centre (JRC), Ispra, Italy; b European Commission, DG for Regional and Urban Policy, Brussels, Belgium; c The Productivity Institute, Alliance Business School, University of Manchester, Booth Street West, Manchester M15, United Kingdom; d University of Calabria, Department of Economics, Statistics and Finance “Giovanni Anania”, Ponte Bucci, cubo 1C, Arcavacata di Rende (CS), I-87036, Italy

**Keywords:** Well-being, Regional policy, Dose-response function, Generalised propensity score

## Abstract

Enhancing the well-being of its citizens is the central remit of the EU's regional policy, but as yet, there is no analysis of the effects of EU regional policy on local well-being. The aim of this paper is to examine this relationship. To do this, we define a novel regionalised well-being measure and we exploit a dataset on regional expenditure in a continuous treatment framework. Based on both parametric and semi-parametric approaches, our analysis demonstrates that the EU regional development policy does influence regional well-being differently from GDP. We find evidence of a linear monotonic response of well-being growth to total transfers, although this effect varies according to the time lag considered and the level of development of the region.

## Highlights

• This paper examines how the world’s largest place-based economic development policy is related to regional well-being
• Well-being is measured by a novel composite indicator defined at the regional level in EU15.
• A dose-response function for different funding intensities is estimated both in a parametric and semi-parametric framework
• EU regional development policy does influence regional well-being differently from GDP.
• The effect varies according to the time lag considered and the level of development of the region.

## 1 Introduction

In recent years there has been a growing interest in the role and effectiveness of place-based economic development policies (Gobillon et al., 2012; Klein and Moretti 2014; Ahlfeldt et al., 2018; Criscuolo et al., 2019; Falck et al., 2019; Ramboer and Reynaerts 2020). Many papers focus on specific policies with specific objectives and in particular countries, thereby making generalisations to other contexts sometimes rather difficult. In this respect, analysing the effects of EU regional development policy can be useful both because it traverses national boundaries and has various policy objectives built into it, thereby allowing for more realistic generalisations across places and themes (Becker et al., 2010, 2012a,b, 2018; Cerqua and Pellegrini 2017; Crescenzi et al., 2020; Ferrara et al., 2017; Pellegrini et al., 2013). The regional and urban development policy of the European Union, known as EU Cohesion Policy, is the world's largest place-based economic development policy operating within a single legal and institutional set-up, accounting for a third of the overall EU budget, and involving many thousands of individual policy actions and interventions (McCann 2015). The overall ultimate goal of Cohesion Policy is that of improving citizens' well-being, especially in the least developed areas (Barca 2009).

However, while there is now a whole suite of papers examining the effects of EU Cohesion Policy on productivity and growth, virtually none of the existing studies has assessed the impact of the policy in terms of well-being. This paper contributes to this lack of empirical evidence, by examining to what extent EU regional well-being is affected by Cohesion Policy funding and how well-being growth reacts to different amounts of policy transfers. Moreover, the study also compares the effects on the promotion of multidimensional well-being to those in terms of GDP growth.

In order to do this, we develop a novel measure of objective well-being for NUTS 2 regions in EU15 based on observable indicators, rather than employing subjective self-reported well-being data, by combining several dimensions of social and economic progress and we exploit a recently-published dataset on regional modelled expenditure (Lo Piano et al., 2017) in a continuous treatment framework. The response of our well-being outcome to regional policy transfers is assessed by using both a parametric and a semiparametric approach, allowing us to identify whether there is evidence of an optimal desirable treatment intensity (Hagen and Mohl, 2008) and/or a minimum necessary level of regional transfers required in order to induce well-being growth effects. To the best of our knowledge, none of the existing research has assessed the link between well-being indicators and EU Cohesion Policy in a continuous treatment framework, and the policy relevance of these issues has recently increased dramatically due to the tighter budget of the European Union and the debt crises of many Member States (Becker et al., 2012b).

In recent decades, the attention to the multidimensional nature of people's well-being and quality of life has also gained significant momentum in the agenda of policy makers and international institutions as well as in public and academic debates^1^ (Fleurbaey, 2009; Stiglitz et al., 2009). In particular, Stiglitz et al. (2009) argue that to measure economic performance and social progress, it is important to focus not only on production outcomes but also on several aspects which are crucial for people's well-being and quality of life, such as health, education, culture, leisure, environment, personal security and social relations. Yet, on methodological grounds, there is still no consensus on the best approach to follow (Dialga and Thi Hang Giang, 2017; Hagerty and Land, 2007; Saisana et al., 2005). Moreover, self-reported well-being scores are often conceptually and empirically inconsistent with utility (Bond and Lang 2019; Anderson et al., 2016; Odermatt and Stutzer, 2019), and nor are they necessarily affected by specific policies aimed at determining an improvement of living standards (Galiani et al., 2018). Indeed, these problems are especially acute at the local and regional levels where local peer-group effects can confound absolute effects in diverse ways (Rijnks et al., 2019).

Our composite indicator approach also complements other approaches which seek to uncover the well-being effects of place-based policies, in particular in Europe (Blouri and Erlich, 2020; Ahlfeldt et al., 2020; Henkel et al., 2021). Standard spatial equilibrium models do not have directly observable measures of utility or utility changes, but rather they infer these indirectly via changes in factor prices and mobility in the context of model assumptions. The inferred utility values are derived via model inversion in the context of the model estimations, although the accuracy of these inferences depends on the veracity of the assumptions underpinning the spatial equilibrium models. A recent set of papers (Redding and Rossi-Hansberg 2017; Desmet et al., 2018; Blouri and Erlich, 2020; Henkel et al., 2021; Ahlfeldt et al., 2020) construct spatial general equilibrium or dynamic models and then via model inversion determine amenity values reflecting utility net of wages. Assuming that the structural fundamentals are stable and invariant to the policy interventions, then these models also allow for counterfactual calibration for policy analysis (Redding and Rossi-Hansberg 2017), with various papers finding well-being increases linked to place-based policy interventions in Europe (Blouri and Erlich, 2020; Ahlfeldt et al., 2020; Henkel et al., 2021).

In our framework, utility is not inferred via the model and no assumption is made as to the agents’ behaviour and model structure. Our well-being indicator could be considered as a direct measurement of those factors that qualify as amenities in a standard spatial equilibrium model and that determine to what extent a place is desirable (Redding and Rossi-Hansberg 2017). However, the model flexibility and the more parsimonious data requirements come with a downside in terms of subjectivity in the selection of variables, their weighting scheme and aggregation method. To reduce arbitrariness, we therefore select observable and measurable variables that the literature has identified as having direct effects on well-being. We then examine the effect on well-being outcomes of place-based policy interventions for the major influences which policy was targeting.

Our paper also differentiates from the approach based on subjective measures of well-being that attribute a score to individuals’ utility value. In this context, the relationship between utility and well-being appears somewhat controversial, as the latter could be both an input of the utility function and a measure, albeit poor, of the utility level. Along these lines, Glaeser (2007) argues that using happiness to proxy utility might yield misleading conclusions to assess the ultimate goals of public policies.

On the expenditure side, Cohesion Policy operates under the umbrella of the EU-wide (Europe 2020) development agenda of smart, sustainable and inclusive growth, an analogue to the OECD growth agenda of stronger, cleaner and fairer economies.^2^ As such, moving beyond just GDP, as well as enhancing productivity and innovation, through its funding schemes Cohesion Policy also underpins actions aimed at combating poverty, increasing social inclusiveness and enhancing sustainability, in which indicators of social progress are explicitly taken into account as well as purely economic measures in any policy evaluation (Barca and McCann, 2011; Dhéret, 2011; European Commission,^2008^, ^2014^). In particular, Cohesion Policy encompasses different funding streams (which are technically termed as the European Structural and Investment Funds) having different priorities. The European Regional Development Fund (ERDF), the economically oriented Cohesion Policy component, focuses on infrastructure provision, support for business innovation and growth, and environmental upgrading. The European Social Fund (ESF) is instead, more socially oriented and therefore focused on various dimensions of people's well-being and quality of life, including improvements in employment and education opportunities, especially for the most vulnerable people at risk of poverty. The ERDF and the ESF are the two primary funding programmes of Cohesion Policy^3^ and are at the core of our analysis.

The funding allocation mechanism makes the eligibility criteria for ERDF funding clearly demarcated geographically according to their GDP per-capita levels via the ‘Objective’ status of the regions, with Objective 1 regions being the least developed regions, which are eligible for the largest funding allocations. These spatial discontinuities in the policy programming rules have allowed researchers to use binary models and regression discontinuity approaches for evaluating the GDP-related effects of the policy (Becker et al., 2012a, 2018; Ferrara et al., 2017; Pellegrini et al., 2013; Ederveen et al., 2003 and Ferrara and Nisticò, 2016 for a survey). In the period 2000–2006, all regions outside Objective 1 were eligible for ESF funding under Objective 3. In addition, areas facing structural difficulties (Objective 2) and transition regions received ERDF and ESF funding.^4^ As a result, the ESF differs from the ERDF, in that the ESF investments cover all EU regions, both more and less developed, thereby making all of the discontinuities-types of approaches not applicable for analysing the role of the ESF. To overcome this issue, we rely on a continuous treatment framework, measuring treatment intensity with the sum of transfers a region receives under ESF and ERDF. Furthermore, to provide a more nuanced picture, we also estimate the model by considering the two funds separately and their interaction, albeit with the limitation that they might be not independently distributed.

Our findings demonstrate that regional policy transfers do indeed influence regional well-being and GDP, but the ways in which they do this and the required funding intensities differ markedly between the two outcomes. In particular, we observe a linear monotonic relationship between funding transfers and annual well-being growth whereas the response in terms of GDP is more hump-shaped. However, when accounting for some delayed effects of the policy, well-being growth also exhibits decreasing marginal returns.

The rest of the paper is organized as follows. The next section discusses different approaches to well-being measurement and illustrates the data and the empirical design, both on the construction of the EU regional well-being index, and the econometric strategy to estimate the dose-response function, parametrically and semi-parametrically. The third and fourth sections present the results and some robustness checks, respectively, while the fifth section concludes.

## 2 Data and methods

Our analysis is based on a regional panel dataset of about 201 NUTS 2 regions in the EU-15 over the period 2000–2013, combining data on regional well-being and Cohesion Policy transfers. Information on regional Cohesion Policy expenditure is gathered from a recently published DG Regio database covering the period 1989–2014 where raw expenditure data is modelled and harmonised (Lo Piano et al., 2017), while data on well-being dimensions is extracted from Eurostat regional statistics. We focus on expenditure data related to the 2000–2006 programming period of the Cohesion Policy to avoid distortion due to accounting and reporting inconsistencies between different EU multiannual financial frameworks, as well as to have sufficient coverage in terms of well-being data.^5^ We exclude the EU-13 countries that joined the EU in 2004 because they were only partially exposed to the transfers in the period under analysis.^6^

As far as Cohesion Policy expenditure is concerned, we focus on the sum of transfers under the ESF and the ERDF. On the one hand, we expect ESF transfers to be an important determinant of regional well-being as they envisage interventions mainly targeted at individuals, such as active labour market programmes or lifelong learning activities. On the other hand, we expect ERDF to affect well-being by providing better infrastructure and commercial endowments such as transport facilities and school construction, along with business innovation and entrepreneurship incentives.

The scope of our analysis is not only to estimate a dose-response function of well-being to Cohesion Policy transfers but also to uncover potential differences with respect to the dose-response function obtained with GDP growth as an outcome. In this framework, cohesion expenditure is a continuous treatment that affects all the regions but with heterogeneous intensities.

The remainder of the section briefly discusses some approaches to well-being measurement, and then it defines the well-being indicator and finally describes the empirical strategy to estimate the response to Cohesion Policy transfers.

### 2.1 Measuring well-being

The recent academic and policy debate is increasingly focusing on the effect that economic progress has on people's well-being and living conditions. The publication of the Report by the Commission on the Measurement of Economic Performance and Social Progress (Stiglitz et al., 2009) has emphasized the need to analyse progress in a more comprehensive way that goes beyond the standard GDP statistics. Indeed, GDP offers unrivalled advantages in terms of data coverage and comparability, but it represents a weak measure of the quality of life.

The public economics literature has mainly investigated well-being within the relationship between economics and welfare, as a proxy of utility. Utility maximization is considered the ultimate goal of individuals' optimization strategies and a measure of their well-being. This approach combines individual behaviour with public policies' objectives that also seek to improve people's well-being (Gerritsen, 2016). However, in this framework, well-being is primarily conceived as a subjective measure, which is often represented by a categorical variable in a 0–10 scale and answering the survey question “All aspects considered, how happy/satisfied do you feel about your life?” Subjective well-being measures have been widely used to assess the impact of specific events or policy changes (Anderson et al., 2016; Danzer and Danzer, 2016; Deaton, 2018; Dolan et al., 2019) or to monitor social progress (Dolan and Metcalfe, 2012; Stiglitz et al., 2009; OECD, 2013).

Despite its widespread diffusion in welfare studies, subjective well-being is considered a poor proxy of utility (Bond and Lang 2019), which might lead to biased estimates if there is a systematic error term relating to utility and subjective well-being. Moreover, in the long-run individuals might adapt to their new life conditions (mean-revert) and, hence, this might affect the self-reported subjective well-being, thereby distorting marginal differences computed over time (Anderson et al., 2016). The existence of this systematic error term is proved by Odermatt and Stutzer (2019) for the specific case of changes in circumstances due to major life events. More generally, the assumption is that individuals do not compare themselves to an absolute standard, but in reference to a relative standard, which varies over time (Kievit et al., 2010). Indeed, this is especially problematic in a geographical setting (Rijnks et al., 2019) where relative local peer-group effects can override absolute effects in complex and differing ways, depending on the local context. Therefore, a measure of subjective well-being would not suit the purpose of our analysis because it may well be unresponsive to an improvement of objective living standards (Galiani et al., 2018), which is what EU regional policy strives for.

In spatial equilibrium models, utility is often endogenously derived from people's location choice and precise assumptions are framed within the model structure. In this perspective, quality of life stems from territory-specific characteristics generating shifts in individuals' utility; thus, location choices reveal local features that contribute to maximising utility and well-being. In a neoclassical framework, with no migration costs, homogeneous workers with spatially invariant utility move in a costless manner from one place to another; hence, differences in the value of amenities between two locations are expected to be offset by differences in real wages and rents, giving rise to perfect spatial arbitrage (Rosen, 1974, Rosen, 1979; Roback 1982; see Redding and Rossi-Hansberg 2017 for a survey). However, in reality, workers have heterogeneous location preferences and face both monetary and non-income migration costs, therefore arbitrage is likely to be only imperfect (Ahlfeldt et al., 2020).^7^

Our analysis generates complementary insights from a different perspective and leads to an approach that can be readily replicated in different contexts with fairly parsimonious data requirements. Based on the principles of Stiglitz et al. (2018), we move beyond GDP and investigate data on factors which are known from worldwide evidence to affect well-being. One of the insights from the well-being literature that might fruitfully be incorporated into more traditional spatial economic models is the notion that greater social equality in outcomes and levels of participation are associated with higher utility. Nordic countries, and other European and Australasian countries with relatively low levels of inequality, consistently rank very high in well-being and happiness rankings. None of these countries has very large cities, and most of them are interregionally relatively equal. This also suggests that some of the well-being-related aspects of cities may attenuate well before the scale-related economic advantages of cities do. Similarly, in some circumstances the amenity aspects of cities maybe more important than the density-related and scale-related productivity advantages of cities. Importantly, well-being reflects responses to a composite bundle of influences, which vary by location.

Over and above personal and family characteristics, international survey-based evidence (OECD 2018) confirms that there is a range of variables which affect well-being. Our composite index is therefore constructed on comparable and standardised data for those well-being-related variables which most closely correspond to the intended objectives of the policy. As such, we focus on economic development, labour participation, skills and education, health, and regional attractiveness. There are other issues such as civic participation, crime, and work-life balance, which are known to influence well-being (OECD 2018), but these were not primary domains for EU regional and urban policy interventions for the period we examine.

Nonetheless, some of these variables may display interaction effects. On the one hand, higher human capital tends to be associated with higher incomes, and higher-income people tend to have better employment trajectories and tend to live longer and in higher amenity areas. On the other hand, in high amenity environments, people may be willing to accept lower incomes (Graves 1980, 1983), and this wage-compensation effect may be even more pronounced in places with both high amenities and high knowledge spillovers (Glaeser et al., 2001). In other words, these interaction effects may exhibit either complementarity or substitutability properties in different contexts. Composite indices allow for these various types of substitution or complementarity effects, and the construction of our composite well-being index is based on international best-practice principles (OECD, 2008, 2018).

If well-being is highly correlated with utility, and place-based interventions increase well-being in those places where the actions take place in a manner which is related to the scale of the interventions, then in principle, the place-based policy interventions should alter the general spatial equilibrium conditions. This in turn should engender factor mobility responses. In particular, we might expect in-migration into those regions experiencing increased well-being, and capital inflows ought to follow if increased housing and business investment opportunities are also associated with these labour inflows^8^ (Glaeser 2007; Moretti 2011). A more thorough discussion on how multidimensional well-being measures can be framed in a Rosen-Roback model (Sheppard, 2013) is reported in Section A.1 in the Appendix in the supplemental online material.

### 2.2 A composite well-being index

The literature on the appropriate measurement of objective well-being has grown enormously in the last decade (see Ferrara and Nisticò, 2019 for a survey) with a corresponding increase in the number of dedicated initiatives by international organisations and national institutes of statistics that have mainly steered the design and publication of specific datasets. The vast majority of these initiatives remain somewhat ad hoc in that they have not made the final step of delivering a composite indicator that can be used for empirical and analytical work. This leaves still open the issues related to the absence of a standard composite measure of well-being for all EU NUTS 2 regions as well as the recurring problem of subjectivity in the choice of the relevant dimensions to consider and their respective weights.^9^

Hence, to provide a more accurate analysis of well-being responses to Cohesion Policy transfers we define a composite well-being indicator (*WB*) for the European NUTS 2 regions over the period 2000–2013. This would be one of the first attempts to measure multidimensional well-being at the regional level with an EU-wide coverage. We propose a composite well-being index which covers five economic and social progress dimensions: economic condition, education, health status, labour market participation and regional attractiveness. As suggested by recent literature focused on industrialized countries (Marchante and Ortega, 2006; Stiglitz et al., 2009) our composite index includes a wider number of well-being components than the Human Development Index (HDI) proposed by UNDP. Moreover, following Hagerty and Land (2007), Ogwang and Abdou (2003), and Sharpe and Andrews (2012) we apply the system of equal weights (EW), resulting in the lowest level of disagreement among the large variance in individuals’ weightings.

Our baseline indicators are then scaled into the (0, 1) interval with a min-max normalisation (Marchante and Ortega, 2006; Mazumdar, 1999; Ferrara and Nisticò, 2013):

$$sY_{i}^{t}=\frac{\left(Y_{i}^{t}-Y_{\text{i }min}\right)}{\left(Y_{imax}-Y_{imin}\right)}$$

where $Y_{i}^{t}$ is the value of the variable *i* in year *t*; $Y_{imin}$ and $Y_{imax}$ are the minimum and the maximum value of the variable in the period under consideration, respectively. $sY_{\text{i}}^{t}$ assumes values between 0 and 1. As a final step, all the *i-th* variables are aggregated into our well-being indicator by the arithmetic mean and eventually the indicator itself is again min-max normalised.

Our composite index is consistent with the latest thinking regarding the construction of composite indicators without facing the various methodological problems associated with subjective well-being measures. Furthermore, it captures the well-being performance of weaker regions in comparison to the most prosperous ones, the enhancement of which is precisely the objective of Cohesion Policy.

We consider per-capita disposable income as a proxy for material living conditions, determining people's ability to satisfy their needs and aspirations, defined as one of the essential components of well-being (OECD 2020).

Education positively influences a variety of aspects of people's lives (Michalos, 2011) including public health, environmental care, greater social cohesion and civil rights protection (Acemoglu and Angrist, 2000; Hanushek and Woessmann, 2008; Lochner and Moretti, 2004; Sianesi and Van Reenen, 2003). Moreover, highly educated individuals are largely attracted by locations characterised by high amenities (Buettner and Janeba 2016; Diamond 2016; Falck et al., 2011; Moretti 2004). In our composite index, we measure education as the share of the population (aged 25–64) that achieved a level of education between 3 and 8 (ISCED).

Health status is represented by a standard composite index based on a linear combination of life expectancy at birth and the infant mortality rate^10^. Health is among the most important factors that people indicate as influencing their well-being and the most common dimension included when constructing composite well-being indicators (OECD 2013). Life expectancy at birth represents the standard procedure for measuring the length of human life used by the UNDP (UNDP 2019). The infant mortality rate measures deaths during the first year of life per 1000 live births and is an important measure for policies aimed at improving social health, hygiene and nutrition.

People's employment opportunities is one of the most relevant quality of life dimensions. This is also confirmed in the spatial models literature by which “identifying the reasons why agents locate in a particular place necessarily requires taking a stand on the opportunity they have to move across locations in search of better living and work opportunities” (Desmet et al., 2018, p. 905). We define labour market conditions along three dimensions: female participation in the labour market, the (reciprocal) share of youth unemployment and of those that are neither employed nor in education (NEET).^11^

Finally, regional attractiveness is represented by the reciprocal of population density and an indicator of touristic flows given by tourist arrivals over tourism capacity in terms of bed places. The potential adverse impact of population density on the quality of life is a proxy for congestion (Cramer et al., 2004; Fassio et al., 2013), which is typically treated as a negative externality in most urban economic and economic geography models. A large concentration of population in a location indeed negatively influences its amenities (Desmet et al., 2018). Conversely, regional attractiveness is also linked to the concept of competitiveness and quality of the tourism experience: measuring visitor numbers is a direct and objective way to assess tourism success (Dupeyras and MacCallum, 2013).

In a standard Rosen (1979) - Roback (1982) framework, higher regional well-being would be associated with both higher labour inflows and higher land prices. In a similar perspective, studies in regional economics describe net migration as an indirect measure of local well-being or amenities (Faggian et al., 2012; Gollin et al., 2021) or estimate regional utilities by using migration data (Nakajima and Tabuchi, 2011), under the assumption that individuals reveal their preferences by moving to more desirable locations as they “vote with their feet” (Tiebout 1956; Douglass and Wall 1993). Spatial equilibrium models consider migration dynamics, adjustments in land rents and housing prices as key elements to measure the quality of life.

To test the validity of our indicator against these hypotheses we consider the levels of net migration and the nominal national house price indices as proxies for labour inflows and land prices, respectively. Ideally, one would need to assess worker differences across regions, rents or house prices and differences in house quality across regions. Unfortunately, these data are not available over a regional EU-wide scale and are not harmonised to allow cross-country comparisons.

**Fig. 1:**
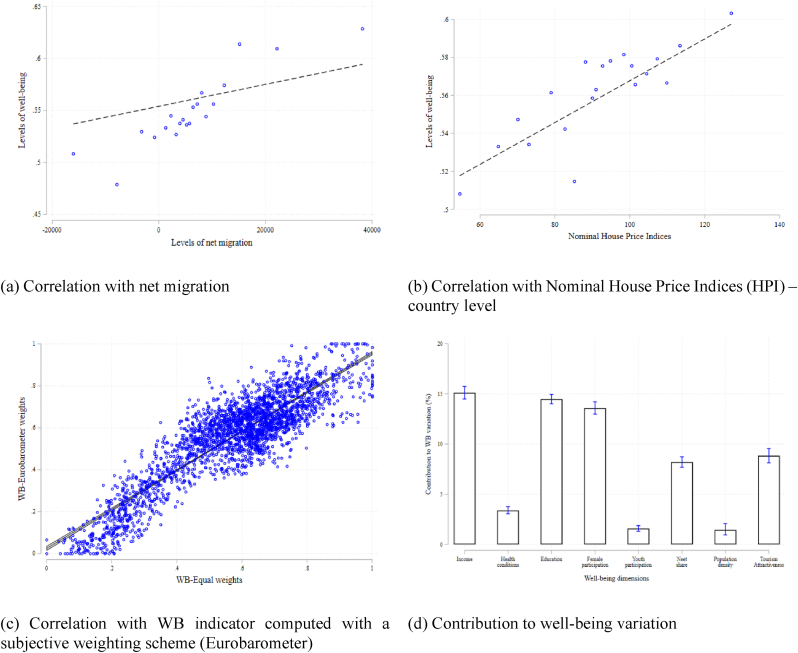
Sensitivity checks on the well-being index. Notes: All results are based on a longitudinal dataset covering the period 2000-2013. Only for panel (b), the analysis is at the country level due to a lack of HPI data at the regional level. Panels (a) and (b) show binned scatterplots absorbing country fixed effects from the x and y-variables before binning and plotting. The blue dots are the median values of the x-axis and y-axis variables within the equal-sized bins grouping the two variables, whereas the dashed linear fit line is estimated using the underlying data, not the binned scatter points. Panel (c) combines a non-binned scatterplot with the linear fit line of the y-axis variable on the x-axis variable, and the shaded grey area is the respective 95% confidence band. Figure (d) plots the Owen value decomposition for regional well-being, and the blue vertical whiskers are the 95% confidence intervals.

The binned scatter plot in Fig. 1 (panel a) suggests that our well-being index (in levels) is positively associated with the levels of net migration, which is a general estimation of the net migration based on the difference between population change and natural change between two dates (in the Eurostat database it is called net migration plus statistical adjustment).^12^ In a similar vein, we then explore the association between our regional well-being indicator and the national Nominal House Price Indices (HPI) published by the OECD.^13^ HPI is a nominal index that measures the prices of residential properties over time, for each country, covering the prices for the sale of newly-built and existing dwellings.^14^ Fig. 1 (panel b) plots the correlation between well-being growth and the housing cost variable, showing a positive coefficient of about 0.011, statistically significant at the 1% level. This evidence, albeit suggestive, points in the expected direction of a positive association between amenities and house prices.

Migration responses and adjustments both in local prices and in land rents play a crucial role in determining the efficiency of spatially targeted transfers (Blouri and Erlich, 2020). Using a natural experiment from Germany, Ehrlich and Seidel (2018) show that a large-scale transfer programme in a well-defined geographical area, designed to stimulate economic development, led to higher income per square kilometre in the treatment area and, at the same time, raised population density, with no indication of a decline in the long term in both variables. Einio and Overman (2020) document employment displacement effects from untreated to treated areas in a counterfactual study regarding the impacts of a significant place-based intervention in England to increase employment and productivity in backward areas. In a study conducted on the EU15 Member States over the period 1986–2004, Egger et al. (2014) report that an increase of international transfers to finance infrastructures in regions which were performing worse than the EU average, such as structural fund expenditures, lead to a reduction of bilateral net migration flows across borders.

As an additional sensitivity check, we demonstrate that our indicator is robust to a subjective weighting scheme derived from a Eurobarometer survey. Quality of life is addressed in the Eurobarometer in two ways: (1) as time series indicators regarding the general judgment of living conditions and (2), intermittently, the Eurobarometer includes special modules on quality of life, particular life domains, and related policies. The Eurobarometer wave EB52.1 (Eurobarometer, 2000) was in line with our goal to find a survey where participants were asked to value each domain of quality of life in a discrete scale, thereby allowing for the deriving of the relative subjective weights for the components to be aggregated in our well-being composite index.^15^ The timeframe of the survey fits our analysis as it precedes the transfers’ disbursement and helps to avoid endogeneity issues. For each dimension of our well-being index, we calculate the weights based on the survey answers and we then compute our composite indicator as a weighted average.^16^ As Fig. 1 panel (c) shows, results point to the existence of a strong and significant correlation (0.9) between the original WB index and the one using weights drawn from the Eurobarometer survey (EB-WB). However, the indicator obtained applying equal weights remains our preferred specification to avoid the limitations that subjective weighting might imply (Sharpe and Andrews, 2012; European Commission- JRC, 2008) and the distortions due to self-reported measures of well-being (Anderson et al., 2016; Kievit et al., 2010).

Given the multidimensional nature of our well-being index, it is worth exploring the extent to which each dimension contributes to explaining the variability of the composite indicator. To this end, we adopt the approach proposed by Huettner and Sunder (2012) relying on the estimation of the Owen value, a generalisation of the Shapley value decomposition.^17^ In our case, we regress the composite well-being indicator on its components accounting for groups defined over countries and years.^18^

Fig. 1 (panel d) plots the Owen value decomposition for regional well-being, with 95% confidence intervals, based on 5000 bootstrap replications. According to our results, about one-third of the explained variance can be attributed to country dummies, whilst the contribution of year dummies is only marginal (1.71%). The group including all well-being dimensions accounts for approximately two-thirds of the overall variance, reinforcing the notion that this is the most important one. Within this group, each dimension is statistically significant at least at 5% with disposable income, education and female participation playing a larger role (>10% of R^2^). Non-negligible is also the contribution of the reciprocal of Neet share and regional attractiveness (>8% of R^2^) and health conditions (>3.5%), whereas population density and touristic attractiveness have a more marginal role (around 1.5% of R^2^). However, for these two dimensions, the absence of overlap between confidence intervals is not verified, hence suggesting that no generalisations on the difference in importance could be drawn.

**Fig. 2:**
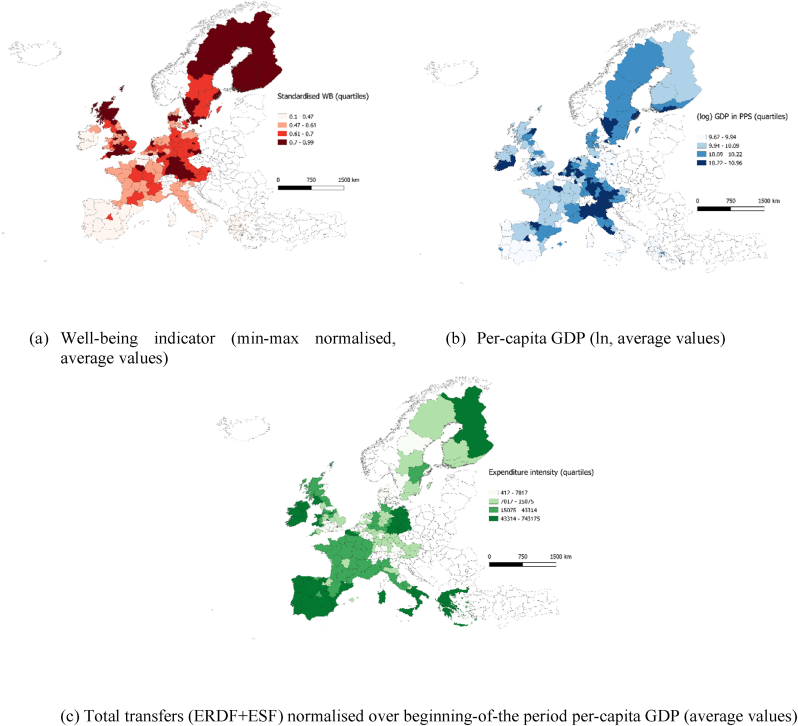
Geographical distribution of well-being, per-capita GDP and Cohesion Policy expenditure.

Fig. 2 plots the geographical distribution of the quartiles of our well-being indicator *WB* (panel a), (log) per-capita GDP in PPS (panel b) and Cohesion Policy expenditure over per-capita GDP in PPS (panel c). The maps show that there is no perfect overlap of regional performance in terms of well-being and per-capita GDP, albeit the two factors have a correlation coefficient of about 0.6**.** Panel (c) plots the distribution of average total expenditure (ESF + ERDF) and confirms the widely debated centre-periphery paradigm, with a higher concentration of the funds in peripheral areas (dark-shadowed) and a lower intensity in the central (core) regions. Panel (c), hence, shows the reverse picture with respect to panels (a) and (b) darker shadowed in the central area.

More in detail, Table 1 reports the descriptive statistics for the main outcome and expenditure variables we employ in our analysis. Our sample consists of 2639 region-year observations, of which 825 were eligible for Objective 1 funding in the 2000–2006 programming period. This amounts to 31% of the whole sample, confirming that the regions more intensively treated by Cohesion Policy do not represent the majority of our sample. By looking at the expenditure data,^19^ it comes out that ESF and total expenditure have a similar mean value (approximately 16 million euros). Not surprisingly ERDF mean is slightly higher (about 18 million euros) but still comparable with the others and in line with the funding interventions’ mechanism. As regards the outcome, well-being growth ranges from 0.89 for the annual rate, to 1.83 for the growth rate computed over two years to 2.23 when considering a three-year interval. Per-capita GDP exhibit higher growth rates than well-being independently of the time window considered.^20^

**Table 1:** Descriptive statistics.

Variable	N	min	max	mean	sd
g(WB) - annual	2437	−100.00	119.60	0.89	11.66
g(WB) - over two years	2236	−100.00	80.85	1.83	14.07
g(WB) - over three years	2036	−100.00	102.20	2.23	15.60
g(GDP) - annual	2405	−17.08	20.24	1.85	4.13
g(GDP) - over two years	2204	−21.40	21.50	3.60	6.46
g(GDP) - over three years	2003	−32.26	27.95	5.18	8.16
ESF expenditure (norm-GDP pc)	1899	0.00	100.00	59.28	14.70
ERDF expenditure (norm-GDP pc)	1872	0.00	100.00	61.01	15.52
Total expenditure (norm-GDP pc)	1902	0.00	100.00	59.28	14.33
ln (per-capita GDP in PPS)	2606	9.39	11.14	10.10	0.26
Objective 1	2639	0.00	1.00	0.31	0.46

Notes: Our dataset is an unbalanced panel of 201 EU15 NUTS 2 regions over the period 2000–2013. We miss information on the four French overseas departments, the four autonomous Portuguese regions and Spanish regions. Well-being growth rates are computed over min-max normalised indicator values to allow for comparison across years. Per-capita GDP in PPS is expressed in log. Expenditure data are gathered from DG REGIO and are then normalised over the beginning of the period per-capita GDP (in PPS) and min-max normalised to let the treatment vary into the (0–100) interval and to mitigate the size effects due to a different regional economy scale, as also proposed by Cerulli (2015). The variable Objective 1 is a dummy equal to 1 for regions eligible for Objective 1 funding in the 2000–2006 programming period.

### 2.3 Constructing the dose-response function to Cohesion Policy Transfers

European Cohesion Policy targets almost all EU regions with different intensities of transfers. Therefore, the treatment variable may take on several values, in that regions receive different levels of transfers. We seek to investigate how this differential treatment exposure associated with the intensity of transfers has heterogeneous treatment effects on the well-being outcome, by estimating a dose-response function.

The outcome variable is defined as the growth rate of the above-described well-being index (WB) computed annually and both over two and three years. This allows us to assess the effect of the treatment in terms of well-being changes. Furthermore, financial transfers might take some time to affect well-being growth; therefore, we consider the treatment as one-period lagged expenditure levels or as the lagged rolling sum in the two or three preceding years. To dig deeper into regional dynamics we compare the results on well-being with those obtained by considering per-capita GDP (in PPS) as an outcome.

To this end, we adopt both a parametric and a semi-parametric approach, exploiting the advantages and disadvantages of each method. On the one side, the parametric approach allows us to maintain the longitudinal dimension of the data, besides offering accurate point estimates, although there are limitations due to the choice of a specific functional form. To face this issue, we consider both a linear and a quadratic specification, the latter allowing for more flexibility. On the other side, following existing observational studies we adopt a Generalised Propensity Score (GPS) approach, under the *unconfoundedness* assumption. Differently from the existing literature (Becker et al., 2012b; Imbens, 2000),^21^ we rely on Generalised Linear Models to estimate the GPS and we then use kernel methods to get the dose-response function (Bia et al., 2014a)**.**

Our treatment is based on the sum of ESF and ERDF transfers to regions over the analysed period. We also show the results of the model considering the two funding streams separately and their interaction^22^ in order to dig deeper into the effect, albeit with some non-negligible limitations. In line with previous studies (Becker et al., 2012b; Cerqua and Pellegrini, 2018)**,** the expenditure data is normalised over the beginning of the period per capita GDP, to account both for regional population and production size,^23^ and is then scaled into the [0,100] interval, to ease the interpretation of the results (Cerulli, 2015) and to account for regional size effects.

a. Parametric Approach

In order to fully exploit the panel dimension of our data, we first apply a parametric approach that allows us to control for those unobserved factors that might affect the outcome by including a battery of fixed effects. This allows us to identify the average treatment effect on the intensive margins, by estimating via OLS the following baseline equation:

(1) $$gWB_{it}=\alpha +\beta Treatment_{i,t-1}+\delta_{i}+\gamma_{t}+\theta_{ct}+\varepsilon_{it}$$

where $gWB_{it\;}$ is our outcome variable expressed as the well-being growth rate for region *i* at time *t*, $Treatment_{i,t-1}$ is the log of the normalised Cohesion Policy expenditure lagged by one-period. $\delta_{i}$ and $\gamma_{t\;}$ are regional fixed effects and year fixed effects that allow us to account respectively for those unobserved factors which vary between regions and are fixed over time or that vary over time but are common to all regions. However, regions in different countries might still be exposed to different factors over the analysed period, and therefore, to clean the estimated effect from those potential disturbances, we consider an additional term $\theta_{ct}$ which identifies countries' specific linear time trends, accounting for those factors at the country level that vary smoothly over time. $\varepsilon_{it}$ is a stochastic error term. In all the models, standard errors are clustered at the regional level. Furthermore, we estimate the model by considering two different functional forms for the treatment intensity, namely linear or quadratic.^24^

In the quadratic specification, the baseline model becomes:

(2) $$gWB_{it}=\alpha +\beta_{1}Treatment_{i,t-1}+\beta_{2}Treatment_{i,t-1}^{2}+\delta_{i}+\gamma_{t}+\theta_{ct}+\varepsilon_{it}$$

The same models are also estimated by taking per-capita GDP growth rate as an outcome.

Despite providing interesting insights and a straightforward estimation approach, this identification strategy does not account for the possible influence some observable confounders might have on regions, inducing them to heterogeneously react to the treatment received. Furthermore, the parametric approach has a non-negligible drawback aspect nested in its ‘parametric’ nature which requires the functional form to be correctly specified. In order to mitigate this issue and to provide more robust evidence, we also estimate the dose-response function adopting a semi-parametric approach accounting for the effect that different treatment probability units might have according to some observable characteristics (selection into the treatment), as described in the next section.

b. Semi-Parametric Approach: Generalised Propensity Score and Dose-Response Function

Propensity-score methods (Rosenbaum and Rubin, 2006) are extensively used in observational studies to balance the distribution of observable characteristics between treatment and control groups and to compare their outcomes under the *unconfoundedness* assumption, allowing us to eliminate the potential bias in the estimate of the treatment effect. More recently, propensity-score methods have been generalised and applied to those empirical studies where treatment may take on multiple values, hence exposing units to different treatment levels (Imbens, 2000; Lechner, 2010) and on continuous treatments and to arbitrary treatment regimes (Hirano and Imbens, 2005; Imai and Van Dyk, 2004; Bia et al., 2014; Bia and Mattei, 2008; Flores et al., 2012; Kluve et al., 2012).

Our analysis relies on the approach proposed by Bia et al. (2014) which builds on the Generalised Propensity Score (GPS) proposed by Hirano and Imbens (2005) and used to estimate the Dose-Response Function (DRF) of continuous treatment. Differently from Hirano and Imbens (2005), this approach applies semi-parametric techniques to estimate the DRF. This revised approach better suits our research design as it estimates the GPS by fitting the models with maximum likelihood methods that allow the treatment to be non-normally distributed. More specifically, this method requires us: to first estimate the GPS using generalised linear models; then, to impose the common support condition as in Flores et al. (2012) and to assess covariates’ balancing; and finally to estimate the DRF using the estimated GPS by applying their nonparametric inverse-weighting (IW) kernel estimator.

The GPS gives an unbiased estimate of the causal relationship between the assignment level of continuous treatment and the outcome variable. It does not provide explicit information on the effectiveness of the policy, but it estimates to what extent an increase or decrease of the treatment level yields a higher or lower outcome.

To build a quasi-experimental setting, the main challenge is identifying and comparing regions that have similar characteristics but are exposed to different treatment intensities. For each region *i*, we observe a vector of variables *X*_i_,^25^ the treatment intensity *T*_*i*_ and the well-being outcome corresponding to the level of treatment received, Y_i_ = Y_i_ (T_i_). As regards the outcome, we consider here only the annual growth rate. The central assumption is that the assignment to treatment levels is *weakly unconfounded* (Hirano and Imbens, 2005) given the set of observed variables X_i_, $Y_{i}\left(t\right)⊥\;T_{i}|X_{i}$ for all *t ∈ T.*

Therefore, under *weak unconfoundedness*, considering the conditional density of the treatment given the covariates as $\;r\left(t,x\right)=f_{T|X}\left(t|x\right)$*,* the GPS is defined as $R_{i}=r\left(T_{i},X_{i}\right)$, which implies that, within strata, the same value of the score means that the probability that T = t does not depend on the level of covariates. More formally:

(3) $$f_{T}\left\{t|r\left(t,X_{i}\right),Y_{i}\left(t\right)\right\}=f_{T}\left\{t|r(t,X_{i}\right\}\mathrm{for every t \varepsilon T}$$

Hence, any bias associated with differences in the covariates across groups with different treatment levels can be eliminated by using the GPS.

In line with Bia et al. (2014), we estimate the DRF in a two-step procedure: (i) we parametrically model and estimate the GPS and we assess the common support condition and covariate balancing, (ii) we estimate the average DRF, $\mu_{\left(t\right)}$, using a nonparametric IW kernel estimator (Flores et al., 2012). In this approach, the GPS scores are used as a weight to adjust observations for covariate differences.

This analysis allows us to obtain the relationship between different transfers’ intensities and the outcome variables. In terms of policy implications, we are also able to investigate whether there is a desirable level of transfers for short-term counterfactual purposes, whether insufficient transfers may be unable to trigger well-being growth or whether excessive transfers might be wasted or used inefficiently.

## 3 How does regional well-being respond to EU Cohesion Policy Transfers?

Our findings show a multifaceted picture of well-being responses to Cohesion Policy transfers. We first discuss the results of the parametric estimation, followed by those obtained with the GPS.

To mitigate the effect of extreme values for both the parametric and the GPS analysis, we exclude observations falling outside the 2nd and 98th percentiles of the treatment variables distribution. This not only allows us to get rid of outliers but also to consider only regions receiving non-zero transfers, in line with other existing studies (Becker et al., 2012b; Hirano and Imbens 2005).^26^ Moreover, normalisation helps mitigate the size effects due to a different regional economy scale while expressing the transfers in relative terms. The distribution of the treatment variables by doses is plotted by the density functions shown in Fig. A.2. For all the three distributions, the median value is a dose of 60, equal approximately to 17 million euros for ESF, 21 for ERDF and 40 million for their sum. Moreover, we observe that about 90% of the sample receives in absolute terms, more than approximately 2.8 million euro for ESF, 1.5 million for ERDF and 5 million for total transfers.

Table 2 shows the results of the parametric estimations, considering either the annual growth of well-being (Columns 1, 2, 5) or per-capita GDP (Columns 3, 4, 6) as an outcome. In all the cases, the expenditure is normalised over the beginning-of-the-period per-capita GDP in PPS.^27^ The treatment intensity is the normalised value of total transfers in Columns 1–4, whereas Columns 5, 6 show the effect by funding stream, considering ESF and ERDF separately and including an interaction term to roughly measure complementarities or substitutions relationships.

**Table 2:** Parametric estimation of the response function in terms of regional well-being and GDP to Cohesion Policy transfers.

	(1)	(2)	(3)	(4)	(5)	(6)
	Annual g(WB)	Annual g(WB)	Annual g(GDP)	Annual g(GDP)	Annual g(WB)	Annual g(GDP)
Total intensity	0.3275***	0.6045**	0.0340	0.2218***		
	(0.106)	(0.280)	(0.023)	(0.071)		
Total intensity squared		−0.0024		−0.0016***		
		(0.002)		(0.001)		
ESF intensity					0.3391***	0.1071***
					(0.117)	(0.031)
ERDF intensity					0.2460**	0.0970***
					(0.119)	(0.036)
ESF × ERDF					−0.0030*	−0.0015***
					(0.002)	(0.000)
Observations	1622	1622	1597	1597	1609	1463
R-squared	0.17029	0.17087	0.73633	0.73786	0.17123	0.19312
Number of Nuts2	191	191	191	191	192	192

Notes: This table presents the effect on the intensive margins of total (ESF + ERDF) transfers intensity on the annual well-being growth rate (columns 1–2) and per-capita GDP growth rate (columns 3–4). Columns 5–6 look at the effect by single funding stream. Both a linear (columns 1, 3, 5, 6) and a quadratic function (columns 2, 4) of the intensity is proposed. Intensity variable is rescaled over the beginning-the-period per-capita GDP in PPS and then min-max normalised into the (0–100) interval. Across columns, all models include regional and year fixed effects and linear country trends. Expenditure variables are one-year lagged. Standard errors, clustered at regional level, are shown in parentheses. ***, **, and * indicate significance at the 1%, 5%, and 10% level, respectively.

The results point to the existence of a linear monotonic response of well-being growth to total transfers, with a slope of about 0.33 percentage points on the annual well-being growth rate. The effect is comparable in magnitude, albeit not precisely estimated for GDP. Including the expenditure squared term in the model, thereby making the function more flexible, yields similar results in terms of well-being growth as only the linear term is distinguishable from zero. The analyses on per-capita GDP growth depict, instead, a slightly different picture of an increasing function with decreasing marginal returns.

**Fig. 3:**
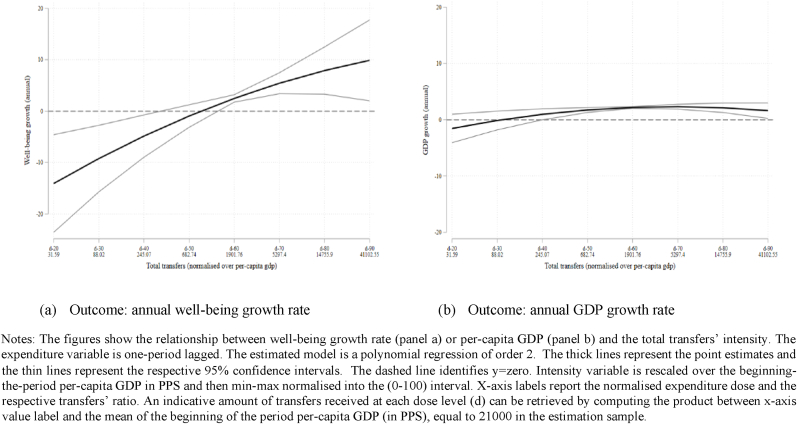
Well-being and GDP response to total transfers (ESF + ERDF).

In other words, switching from a volume of total transfers of about 39 million (dose 60) to 111 million (dose 70) leads to a differential growth in well-being of about 6 percentage points (statistically significant at 5%), which is an increase of about 3% (given that the mean value of well-being growth in the estimation sample is 2.13). When considering the GDP outcome, a similar switch enhances the growth rate by about 2.2 percentage points (an increase of about 1.12%, statistically significant at 1%). These results are plotted in Fig. 3 (panels a and b) that shows the point estimates for different levels of transfers (reporting doses on the x-axis). The figures help us to dig deeper into the heterogeneity of the effect according to the level of transfers received. In particular, we observe that if the average transfers received is lower than 700 times the average per-capita GDP in PPS there might be no significant association with well-being growth. Looking at GDP instead we observe a concave relationship with a maximum in proximity of transfers for 111 million euro (a ratio of 5300).

Furthermore, when considering the interaction between ESF and ERDF transfers we obtain similar results for well-being and GDP outcomes. However, to properly identify the effect of either ESF or ERDF it is necessary to compute the first derivative of the equation with respect to the variable of interest. This translates into deriving from our model the linear combinations $\pi_{ESF}=\;\beta_{1}+\;\beta_{3}\times ERDF$ and $\pi_{ERDF}=\;\beta_{1}+\;\beta_{3}\times ESF$. In both cases, the value of π changes according to the value of the other fund, also offering interesting insights in terms of results interpretation. The coefficient associated to each fund is positive and the interaction negative. The marginal effect of ESF is 0.34 for well-being and 0.11 for GDP at the mean value of ERDF (17.5 million euro). Analogously, the marginal effects of ERDF at the mean value of ESF (14 million euro) are 0.24 and 0.09 on well-being and GDP, respectively. However, this last set of results has to be interpreted with caution given that the two variables might be not independent and identically distributed.

All in all, our results suggest that there is a positive association between Cohesion Policy transfers and well-being growth.

The previous tables and graphs have shown a parametric approximation of the functional relationship between well-being growth and Cohesion Policy transfers. All the findings point to the existence of a positive association with the linear specification of the model for well-being, whereas the analysis on the GDP growth rate shows a slightly different picture as we observe a concave relationship, with higher funds having a positive effect on GDP growth but with decreasing marginal returns, which is consistent with other research (Becker et al., 2012b; Cerqua and Pellegrini 2018).

Taken together, our findings highlight that the effects of Cohesion Policy on growth in regional well-being do not exactly mimic the effects on growth in per-capita GDP. In particular, over the range of observed treatment intensities, the well-being benefits of Cohesion Policy do not display diminished marginal returns akin to those observed for GDP growth. This is likely to be because the well-being index covers a broader set of regional economic development dimensions than just GDP growth, as is consistent with the legal basis of EU Cohesion Policy (Barca 2009; McCann 2015).

However, so far our empirical approach overlooks any observed and unobserved factors that might affect the probability a region has to be treated if there is any self-selection process involved in the treatment assignment. Moreover, the parametric estimation potentially suffers from the adoption of a specific (arbitrary) functional form. To overcome these potential issues, we adopt a semi-parametric approach following Bia et al. (2014). The Generalised Propensity Score (GPS) allows us to remove any bias in the estimate of the dose-response function and to improve regional comparability.

Following Hirano and Imbens (2005) we organise the data into groups of treatment intensity defined accordingly with the deciles of the treatment distribution. The GPS is estimated at the median value of each treatment interval over the common support, improving the comparability of regions with different treatment intensities. Indeed, within the common support, regions receiving a certain amount of funds and with a certain GPS are matched with control regions with similar GPS but a different treatment intensity, thereby avoiding perfect predictability of the intensity given the GPS. The validity of the balancing property is assessed with the likelihood ratio-test.^28^ Once we have eliminated the selection bias, complying with the balancing property, we estimate the dose-response function and the respective treatment effect function (its derivative, here omitted).^29^

**Fig. 4:**
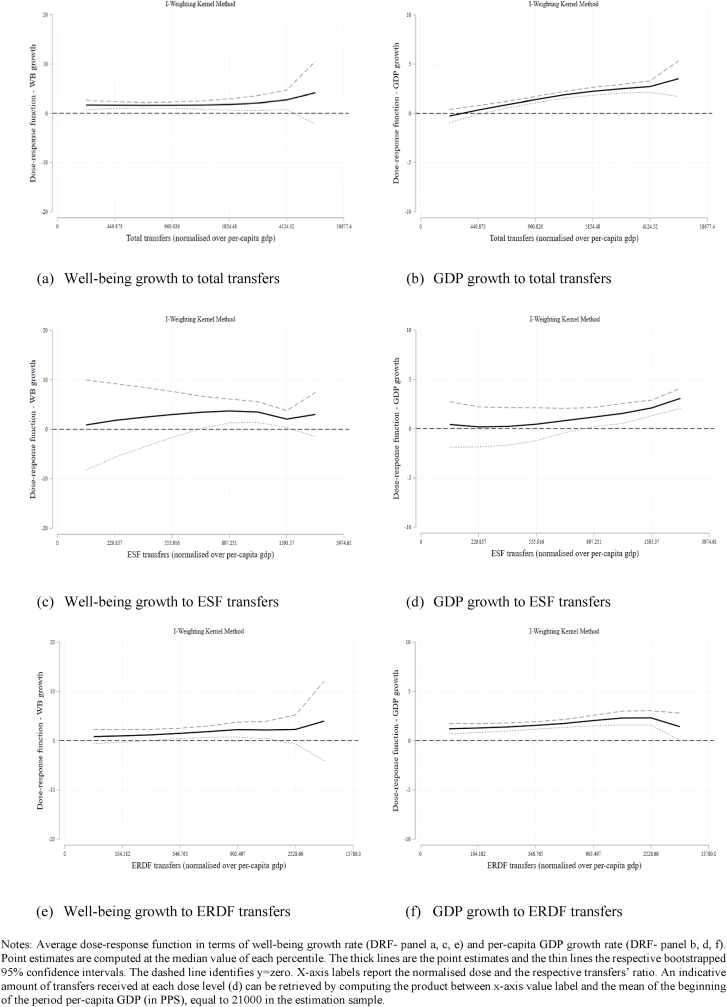
Generalised Propensity Score estimation of the Dose-Response Function (DRF) to Cohesion Policy Transfers (IW kernel method).

In all the panels of Fig. 4, the horizontal axis represents the average ratio between transfers and per-capita GDP (in PPS) at each decile of the distribution of the respective treatment variable (the doses), whilst the y-axis is the respective outcome response. In particular, we are pooling observations throughout the period and we consider the annual well-being growth as the outcome variable and its sub-indicators (Income, Health, Education, Labour market participation and Regional attractiveness) before the policy transfers as observed covariates to estimate the propensity score.^30^ Point estimates of the dose-response function (DRF) are computed at the median value of each group. The thick lines are the point estimates and the thin lines are the respective bootstrapped 95% confidence intervals. To ease the interpretation in terms of statistical significance, the dashed line identifies y = zero.

Panel (a) and (b) in Fig. 4 and Columns (1) and (2) in Table A.3, show the estimate of the dose-response functions to total transfers. Imposing the common support condition drops 204 observations for well-being and 315 for GDP. To assess the balancing property, we consider a Likelihood Ratio test (LR test) which confirms that the GPS balances the covariates (Table A.2). Indeed, both the restricted model that excludes the covariates and the restricted model that excludes the GPS are soundly rejected (p-value is zero). The dose-response function is then estimated using I–W kernel method.^31^ Panel (a) of Fig. 4 shows a slightly convex DRF with a minimum necessary level of transfer in proximity of the seventh decile of the treatment distribution, which is approximately equal to a volume of transfers of about 33 million euro, that is 0.06% of the average GDP in PPS in the estimation sample. This result suggests that for higher values of transfers we should observe an increasing marginal effect on well-being. Panel (b) of the same figure plots the respective DRF in terms of GDP growth rate for which we find a monotonically increasing function, with diminishing marginal returns. These findings mirror what we obtained with the parametric approach and largely validate the results achieved in existing studies (Becker et al., 2012a,b; Cerqua and Pellegrini 2018; Hagen and Mohl 2008).

Panels (c) and (d) show instead the estimated DRF to ESF transfers for well-being and GDP growth, respectively. The balancing property is satisfied and the restricted model without the GPS is again soundly rejected (Table A.2). We impose the common support condition and drop 120 observations for well-being and 18 for GDP. The DRF in terms of well-being is first convex, and then concave. It slightly increases up to a maximum, then decreases, reaches a minimum, and then increases again. However, Table A.3 (Column 3) underlines that these results are not statistically significant at conventional levels. Interestingly, when considering the GDP outcome, the response function monotonically increases for higher ESF transfers, albeit with decreasing marginal returns (Table A.3 Column 4).

On a related note, we investigate the response to ERDF transfers in panels (e) and (f) of Fig. 4. In this case, the common support condition implies dropping 237 observations from the sample for the well-being analysis and 218 for GDP. The balancing property is satisfied; both restricted models are strongly rejected (Table A.2). Fig. 4 shows that the response function is largely similar to that of the treatment based on total transfers, albeit with a less pronounced increase in marginal returns. The DRF of well-being mirrors that to ESF transfers, but is more precisely estimated and the coefficients are significant at conventional levels. The response in terms of GDP growth is again hump-shaped, with a maximum around the eighth decile, with transfers for approximately 53 million euro (which is about 0.11% of the average regional GDP in PPS).

DRF estimates with a semi-parametric approach have broadly confirmed the results obtained in the case of parametric estimation. Overall, these findings provide some insights on the well-being response to place-based policy transfers with an estimate of the Local Average Treatment Effect (as we are focusing on the common support). However, the GPS analysis could not fully overcome issues related to the potential confounding effect that funds might exert on one another and should be interpreted cautiously when considering the two funds separately.

## 4 Robustness checks

Our analysis confirmed that Cohesion Policy transfers indeed play a key role in boosting regional convergence. However, the identification of the right dose might be challenging, especially when the goal encompasses also regional well-being objectives. On the one hand, we have found evidence that the positive marginal effect on GDP fades when transfers are above a certain dose level. On the other hand, well-being dynamics do not fully mirror those of productivity, implying that insufficient interventions might have a decreasing marginal effect on well-being growth.

In this section, we focus on some extensions and sensitivity analysis of the parametric models presented above to better outline specific features of our findings.

### 4.1 Delayed effects of the policy

At first glance, it might be worth investigating how results vary when the effect is evaluated on the growth rates of the outcome variables computed over a two or three-year window, as regions might need some time to implement their development programmes and their effects might take time to materialise. In this case, the expenditure is also expressed as the rolling sum of the transfers in the two or three preceding years.

The results in Tables A.4 and A.5 and in Fig. A.3 confirm those obtained for the annual growth rate. In particular, we find again a linear monotonic function for well well-being, with a larger slope of about 0.44 percentage points on the growth rate over two years. However, when the relationship between total expenditure and well-being is assessed in a three-year window, we find evidence of decreasing marginal returns as the coefficient associated with the squared expenditure term is negative and significant. For GDP growth, we still observe an increasing function with diminishing marginal returns, albeit the squared term is smaller in magnitude and less significant for the expenditure computed as a three-year rolling sum.

The medium-term response to regional policy transfers is in line with the response functions shaped in the shorter-term analysis and reinforces the evidence towards a role of regional policy in boosting well-being growth other than productivity. Moreover, the analysis confirms the existence of different, but not necessarily divergent, paths.

### 4.2 Interdependencies between funding streams

The identification of interdependencies and interrelations between ESF and ERDF that might lead to substitution and complementarities between the two funding streams is beyond the goal of the current study and might not be properly investigated with data and methods at hand. However, Figs. A.4 and A.5 in the Appendix might offer some suggestive insights into this direction providing a more straightforward interpretation of the results showed in Table 2, Tables A.4 and A.5, even if with the stated limitations.

The Figures show the response of either well-being or GDP growth to ESF transfers (Fig. A.4) while keeping ERDF at its mean value (about 21 million euro) or to ERDF (Fig. A.5) at ESF mean value (17 million euro). Panel (a) refers to the annual growth rate, whereas panel (c) and (e) refer to two and three-year windows, respectively. In both figures, panels (b), (d) and (e) show the same results in terms of GDP growth. In all the cases, we estimate a linear polynomial model. The interaction term is always negative and significant, confirming the existence of an interaction between the two funding streams. Moreover, the average marginal effect of ESF on well-being growth is of about 0.6 (p –value is 0.04) after two years and 0.76 after three which is more marked than that for GDP (0.17 in a two-year window and 0.26 in the three-year one). ERDF instead shows an average effect of about 1 percentage points (p-value is 0.002) on the two-year well-being growth and 1.69 (p-value is 0.000) after three years. The effect on per-capita GDP over two years is almost identical, while it drops to 0.31 in a three-year span.

This is in line with the nature of each fund, as the ESF is aimed at supporting jobs and helping people in finding better and fairer jobs opportunities, which could be promoted also with smaller interventions and are less likely to foster well-being growth if not accompanied by wide-enough structural interventions. Conversely, the ERDF is more oriented to the development and structural adjustment of regional economies that might require larger interventions to generate a tangible improvement in living conditions.

### 4.3 Heterogeneity on objective 1 regions

Objective 1 regions account for about 74% of total EU transfers. These are less developed regions, having a per-capita GDP lower than the 75% of the EU average. The effectiveness of Cohesion Policy transfers on their performance has been extensively discussed elsewhere (Ferrara and Nisticò, 2016; McCann, 2015). However, none of the existing studies has considered the impact on well-being, even though enhancing the well-being of its citizens is the central remit of the EU's regional policy. Hence, it is worthwhile investigating whether there is evidence of a differential effect of a different transfers' intensity on the well-being growth in the less developed regions (Objective 1).

To this end, Table A.6 shows the results of the panel estimates that include an interaction term between the intensity variable either as a linear (Columns 1, 3, 5, 7) or a quadratic term (Columns 2, 4, 6, 8) and a dummy variable identifying the Objective 1 status.^32^ In both cases, the effect is given by the linear combination of the coefficients of interest (associated with the linear and quadratic intensity terms) and their interaction with the dummy identifying the Objective 1 regions.

Interestingly, when we consider a linear functional form we find evidence of significant differentials for Objective 1 only for total transfers and well-being growth. Objective 1 regions experience, on average, a higher annual well-being growth to total transfers of about 0.27 percentage points, with respect to non-Objective 1. A positive differential effect of about 0.35 percentage points is also found on the per-capita GDP growth rate in a quadratic specification. The decreasing marginal returns are here again confirmed by the negative coefficients associated with the quadratic terms.

A plausible explanation of this positive differential effect might be nested in the Objective 1 status that gives eligible regions the right to receive higher amounts, therefore boosting the likelihood that these regions are in the domain where DRF exhibits increasing marginal returns. A similar result, also comparable in magnitude, is found for ERDF transfers and well-being outcome.

Our empirical evidence shows that the well-being response to EU policy transfers heterogeneously varies for those regions which are more lagging behind and which show a differential effect with respect to other regions.

Overall, our results suggest that Cohesion Policy intensity matters for the subsequent well-being growth rate. The well-being response to regional transfers is not unambiguous but is related to the amount of transfers and the level of development of a region. The identification of the desirable level of transfers might support the policymaker in the design of the intervention, as well as in terms of public money savings, thereby avoiding the unintended impact that additional policy transfers have a neutral or deterrent effect on the outcome of interest.

## 5 Concluding remarks

This paper examines how an explicitly place-based economic development policy, namely EU regional development policy, is related to the promotion of well-being, by using a novel multidimensional indicator in a continuous treatment framework.

Our composite indicator technique also complements other approaches which seek to uncover the well-being effects of place-based policies, in particular in Europe (Blouri and Erlich, 2020; Ahlfeldt et al., 2020; Henkel et al., 2021). Our analysis indeed generates complementary insights from a very different perspective whereby well-being dimensions are a direct measure of amenities. On the one hand, this approach can be easily replicated in different contexts with a relatively contained data requirement and needs almost no assumptions regarding the model structure and agents' behaviour. On the other hand, it suffers from a non-negligible degree of subjectivity in variables' selection and their aggregation. Coherently with a standard Rosen-Roback model, our well-being indicator is also positively associated with proxies of both labour inflows and land costs.

Despite the fact that well-being promotion is the ultimate goal of the EU Cohesion Policy, there are virtually no empirical studies assessing this relationship. Our paper contributes to this lack of evidence, demonstrating that over large ranges of funding intensity, the EU Cohesion Policy does influence regional well-being in a manner which is consistent with the objectives of the policy. For the most part, higher interventions generate higher well-being growth. However, the ways in which they do this appear to be slightly different compared to the effect on GDP.

In particular, for the combined sum of the funding streams the relationship on well-being growth is broadly linear, while we observe that the significant results in terms of GDP growth all point to the existence of a concave response function that confirms the decreasing marginal returns to investments (Cerqua and Pellegrini 2018; Becker et al., 2012b). Conversely, the benefits of Cohesion Policy to well-being growth do not decrease when GDP growth has diminishing marginal returns, hence suggesting that the broader set of dimensions included in the well-being index react differently to a higher dose of transfers.

## Disclaimer

The scientific output expressed does not imply a policy position of the European Commission. Neither the European Commission nor any other person on behalf of the Commission is responsible for the use that might be made of this publication.

## Funding sources

This research did not receive any specific grant from funding agencies in the public, commercial, or not-for-profit sectors.

## Credit author statement

**Antonella Rita Ferrara:** Conceptualization, Methodology, Formal analysis, Data curation, Writing- Original Draft Preparation, Writing - Review & Editing, Visualization, Supervision. **Lewis Dijkstra:** Resources. **Philip McCann:** Conceptual Framing and Analysis, Writing, Reviewing and Editing. **Rosanna Nisticò:** Conceptualization, Methodology, Writing- Original Draft Preparation, Writing- Review & Editing.

## Declaration of competing interest

The authors declare that they have no known competing financial interests or personal relationships that could have appeared to influence the work reported in this paper.

## Footnotes

1 See Ferrara and Nisticò (2015, 2019) for a survey.

2 See:https://ec.europa.eu/eu2020/pdf/COMPLET%20EN%20BARROSO%20%20%20,007%20-%20Europe%202,020%20-%20EN%20version.pdfand:http://www.oecd.org/corporate/theoecdsefforttohelpbuildastrongercleanerandfairerglobaleconomy.htm

3 The other streams are the much smaller European Agricultural Guidance and Guarantee Fund (EAGGF), the European Maritime and Fisheries Fund, and the Cohesion Fund, which is targeted at countries whose GDP per capita is less than 90% of the EU average, and which includes all EU-13 Accession Countries in central and eastern Europe, plus Portugal and Greece. See McCann (2015) for details of the other funding streams.

4 See 6th Report on Economic, social and territorial cohesion. https://ec.europa.eu/regional_policy/en/information/publications/reports/2014/6th-report-on-economic-social-and-territorial-cohesion.

5 In our records, the last year of expenditure for the 2000–2006 programming period is 2009, so late spenders are also considered.

6 Furthermore, we exclude Ciudad Autonoma de Ceuta, Ciudad Autonoma de Melilla and Ciudad Autonoma dos Azores for their geographical and development distance respect to EU continental regions. For this reason we also exclude the Cohesion Fund which is overwhelmingly focused on the EU-13 countries.

7 Frictional adjustments in the spatial economy are at the core of dynamic spatial models (see, among others, Ahlfeldt et al., 2020; Caliendo et al., 2019; Desmet et al., 2018 and Monras, 2020).

8 In most EU countries, international migration is smaller than interregional migration, and within European countries interregional migration is typically no more than 1% per annum for NUTS2-OECD-TL2 regions.

9 A recent OECD publication (Veneri and Murtin, 2019) proposes a measure of welfare called multidimensional living standards (MDLS) at the regional level. We cannot employ this index because it is available only for two years (2003 and 2012) and only for 64 EU regions. However, the novel indicator of well-being we construct shows also a strong and significant correlation (0.7) with the OECD MDLS indicator, confirming that it is a reliable well-being measure.

10 Specifically, the index is given by health condition = 2/3 × life expectancy at birth +1/3 × (100-infant mortality rate). The different weights allocated to the two variables reflect the greater importance awarded to life expectancy in the UN Human Development Index and mimic the index defined in Marchante and Ortega (2006) and Ferrara and Nisticò (2013).

11 Female participation to the labour market is given by the ratio between the female employment rate (aged 15–64) over the total employment rate (aged 15–64); whilst youth participation to the labour market is given by the reciprocal of the ratio between the unemployment rate of those aged 25–34 and the unemployment rate of those aged 15–74.

12 In different countries net migration including statistical adjustment may, besides the difference between inward and outward migration, cover other changes in the population figures between two consecutive years which cannot be attributed to births, deaths, immigration or emigration.

13 Data comes from the Main Economic Indicators of the OECD and it is available only for the OECD subset of European countries.

14 Unfortunately, this test refers to country level data as HPI is not available at regional level. However, we conducted a similar check, at regional level, considering the OECD regional indicator of housing cost (in % of household disposable income) and the correlation (not reported here) is still positive, albeit with some limitations due to the assumption on individual preferences.

15 This EB wave covers three main domains (Modern Biotechnology, Quality of Life, and Consumers’ Access to Justice). There were 16,082 citizens interviewed in 15 EU countries and in Norway in November–December 1999. In particular, we considered the answers to the question Q15 asking the respondents to identify the three factors contributing more to quality of life in a list of 16 items corresponding to quality-of-life dimensions. The respective answers are included in variables v229-v244 that we used to derive the relative importance of each dimension by country.

16 We group the answers falling in a certain domain, we take the mean and we then compute the average by country. This allows us to have specific subjective weights for each country. Furthermore, we scale the factors to have them summing to 1.

17 First we estimate a full model and we then successively remove independent variables one by one and following a specific ordering. To identify the variable's marginal contribution this approach looks at the differences in the Goodness of Fit associated with the elimination of a variable. The Shapley value of a variable is given by the variable's marginal contribution over all possible orderings. Moreover, the Owen value allows for a decomposition in case of exogenously grouped regressors (Shorrocks 2013) implying that members of a particular group appear only jointly and negotiating a value for the group as a whole.

18 This mimics the estimation of a panel fixed-effects model including both annual and country fixed-effects.

19 To ease the comparison, we report here min-max normalised values.

20 The statistics refer to the full data set at hand. The list of observations dropped by the sample in each specification is reported in Table A.1.

21 Their methodology does not fit our case, because our treatment variable is not normally distributed.

22 Resorting to an interaction variable allows us to account for potential complementarities (or substitutional) relationships between ESF and ERDF on the outcome variable, thereby accounting for the level of the other transfer. A drawback of this specification is a non-straightforward interpretation of the results that we try to address by proposing a graphical analysis. For the GPS estimation, unfortunately an interaction-based treatment variable is not yet implemented, and we account for the level of the other transfer by including, among the matching covariates, a categorical variable identifying the quartiles of the distribution of the other funding stream to which the regions belong.

23 Results hold also when normalisation is run over either GDP or population and are available upon request.

24 In a similar vein, we also estimate $gWB_{it}=\alpha +\beta_{1}ESF_{i,t-1}+\beta_{2}ERDF_{i,t-1}+\beta_{3}ESF_{i,t-1}\times ERDF_{i,t-1}\delta_{i}+\gamma_{t}+\theta_{ct}+\varepsilon_{it}$ to look at the two primary funds separately but only in a linear specification.

25 Our vector of observables (*Xi*) includes the pre-treatment values (year 2000) of the normalised well-being sub-indices, namely: disposable income, health status, education, labour market conditions and regional attractiveness, the log of per-capita GDP (in PPS), a variable identifying the quartiles of the other fund (ESF for ERDF estimation and viceversa) and a dummy for the Objective 1 status.

26 Before the trimming, observations (region-year) receiving zero transfers were 494 for ESF, 397 for ERDF and 193 for their sum.

27 Results hold also with the expenditure variables normalised over beginning-of-the-period population or beginning-of-the-period GDP.

28 Results are reported in Table A.2.

29 Fig. 4 and Table A.3.

30 Moreover, we include a categorical variable defining the quartiles of the other fund and a dummy for Objective 1 regions, so as to increase the likelihood that regions are similar also for transfers received for the other funding stream.

31 Standard errors are bootstrapped with 500 replications.

32 Given that Objective 1 regions are also those receiving the bulk of ERDF transfers, the interaction between the expenditure and this dichotomous variable is already a way to account for the differences in intensity that might exist between the two funding streams.
