# Supplementary material for: The response of regional well-being to place-based policy interventions
Source: Reg Sci Urban Econ. 2022 Nov;97:None. doi: 10.1016/j.regsciurbeco.2022.103830 (PMC9595178; doi:10.1016/j.regsciurbeco.2022.103830)
Supplement: Multimedia component 1 [file mmc1.pdf]

Supplemental online material

Appendix

A.1 Multidimensional well-being in a Rosen-Roback framework

In order to assess the potential well-being-related role of EU regional policy, we can adapt the canonical Rosen-Roback framework. We assume for simplicity that each of the EU regional economies is broadly perfectly competitive in the sense that there are multiple firms generating zero marginal profits and that there are multiple local households consuming the bundles of goods, services and amenities on offer locally. Individual EU regions are typically of the order of 2 million people, so this assumption is broadly realistic. The amenities can be both human-produced urban amenities and also natural amenities. We also know that EU regional policy has numerous simultaneous actions and interventions taking place in each EU region acting variously on the productivity-related challenges facing firms, on the employment, skills and participation conditions facing workers, on the amenity-related features of cities, towns and rural areas, and on the health and quality of life of local citizens.

Given this context, we can use the approach of Sheppard (2013) who adapts the Rosen-Roback framework to consider the interactions between consumption and production enhancements associated with the provision of local public goods.

In Figure A.1**.** (panel a), V represents the indirect utility function associated with consuming local amenities (Roback 1982), while the isoprofit line π represents the trade-offs between land rents r and wages w which face the local firms on the production possibility frontier. As we see, for a given level of firm profitability, any amenity-enhancement policy – which shifts the willingness to pay by households (WTP_h) from V to V' – increases land rents and reduces real wages. Lower real wages for local households act as a compensation for higher local amenities. Meanwhile, in Figure A.1 (panel b) we see that increases in firm profitability allow for both higher wages and land rents, with the shift in the isoprofit line reflecting the willingness to pay by firms (WTP_f). However, at the same time, it is possible that amenity-enhancements, whether they are urban consumption amenities (Glaeser et al. 2001) or natural amenities, may increase local firms' profitability via the attractiveness of a place to higher human capital individuals, as well as via more direct skills-enhancement and economic development policies. In this context, both V and π shift outwards, and while rents unambiguously increase, the effect on wages is ambiguous.

In terms of the Rosen-Roback framework, well-being measures in effect try to capture the values of the areas *OwEr* and in terms of policy interventions, try to assess whether the area $Ow_1E'r_1$ represents an improvement on *OwEr* in the local or regional setting. Wages could increase, decrease or remain unchanged, as depicted in Figure A.1 (panel c); it depends on the shifts in V and π. International research demonstrates that individuals' well-being is associated with a wide range of socio-economic development influences, including wages, employment status and amenities. As such, only looking at wage-income effects may conceal other direct or indirect well-being benefits of regional policy programmes.

The majority of EU regional development funding during the period in question is associated with economic development, skills and employment-related issues, so we might expect that there would be some positive effects on firms' performance (Ferrara et al. 2017), as reflected in wages as well as real estate prices, a situation depicted in Figure A.1 (panel d). In turn, this might be expected to spur labour inflows, given that migration is likely to be associated with local increases in wellbeing (Faggian et al. 2012).

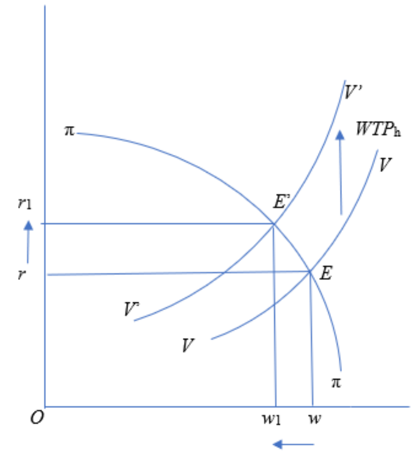

(a) Local Amenity Improvements

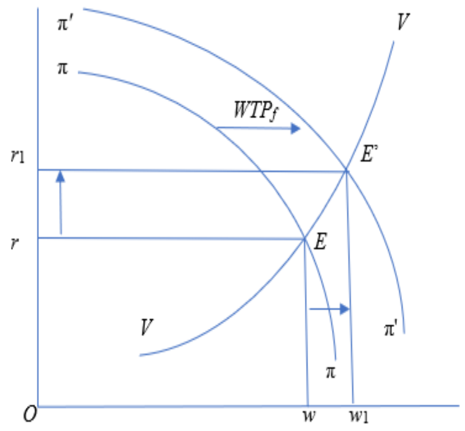

(b) Local Productivity Improvements

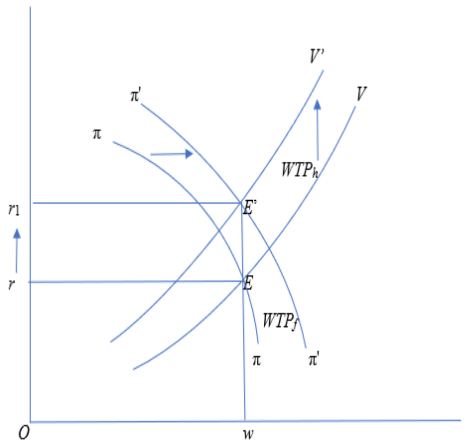

(c) Combined Productivity and Amenity Improvements (No Wage Effect)

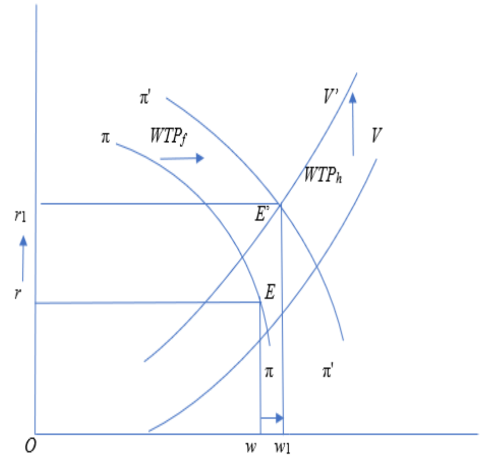

(d) Combined Productivity and Amenity Improvements (Positive Wage Effect)

Notes: The graphs show how isoprofits and indirect utility functions react to productivity or amenity shifts or a combination of them. Wages (*w*) are reported on the x-axis and land rents (*r*) on the y-axis.

Figure A.1 The hedonic effects of a place-based policy

With our composite well-being index, we aim to directly assess evidence on the main domains of regional policy interventions which we know from the literature are associated with well-being. This allows us to develop a proxy for shifts from *OwEr* to $Ow_1E'r_1$ in different regional contexts associated with EU regional policy interventions.

Ideally, we would have real estate data, but unfortunately, for our period of analysis, using real estate data is not possible in this setting. There is no standardised real estate price data which is internationally comparable across EU regions, given the different tax and legal contexts in which such data are set. Therefore, as a proxy for real estate implications, we assess national Nominal House Price Indices (HPI) (Section 2.2).

Inflows of capital are primarily via increased credit to, and investment within, existing firms in situ or locally-based entrepreneurial start-ups, with only a small share attributed to actual firm relocations. Yet, data on capital flows is not available. However, our data does demonstrate that local well-being increases are associated with both labour inflows and higher housing expenditure shares, and these two observations point to an outcome in which local firms' profits are also enhanced, as depicted in Figure A.1 (panel d).[1]

[1] The correlations between our well-being indicator with net migration and housing cost are empirically tested and discussed in section 2.2.

Figures

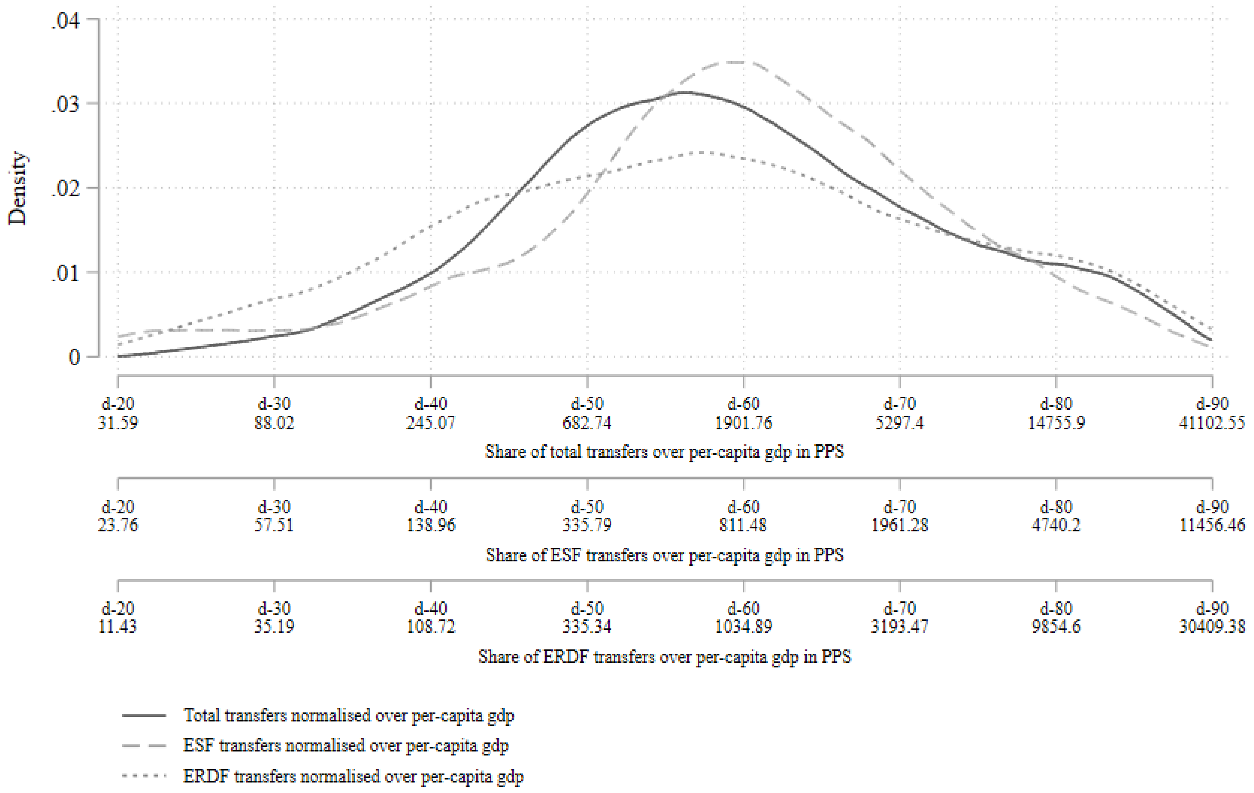

Notes: The figure plots the density functions of the expenditure variables after excluding from the sample observations falling outside the 2nd and the 98th percentiles. Transfers' intensity variables are rescaled over the beginning-the-period per-capita GDP in PPS and then min-max normalised into the (0-100) interval. The thick line plots the density function of total transfers, whereas the dotted and the dashed lines are the densities of ERDF and ESF transfers, respectively. X-axis labels report the normalised dose and the respective transfers' ratio. An indicative amount of transfers received at each dose level (d) can be retrieved by computing the product between x-axis value label and the mean of the beginning of the period per-capita GDP (in PPS), equal to 21000 in the estimation sample.

Figure A.2 Density functions of the expenditure variables

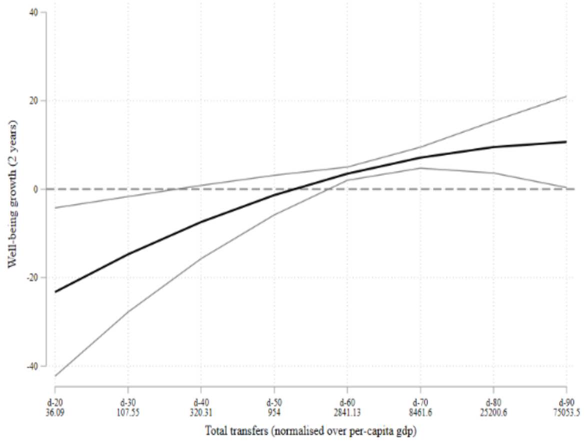

(a) Outcome: well-being growth rate (2 years)

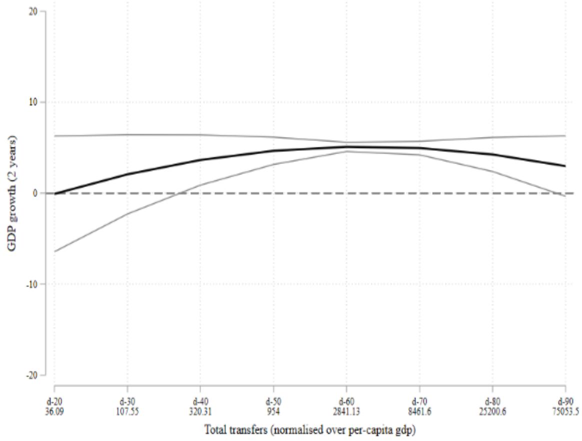

(b) Outcome: GDP growth rate (2 years)

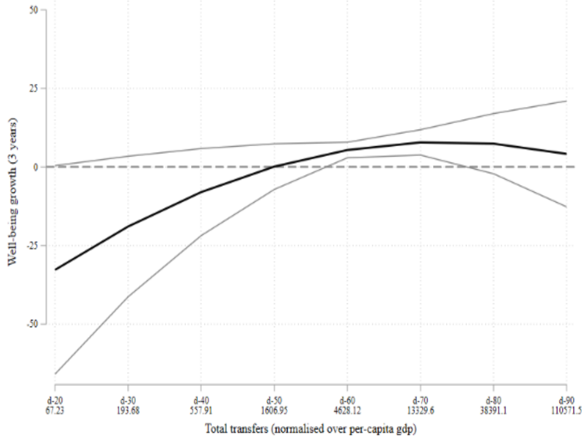

(c) Outcome: well-being growth rate (3 years)

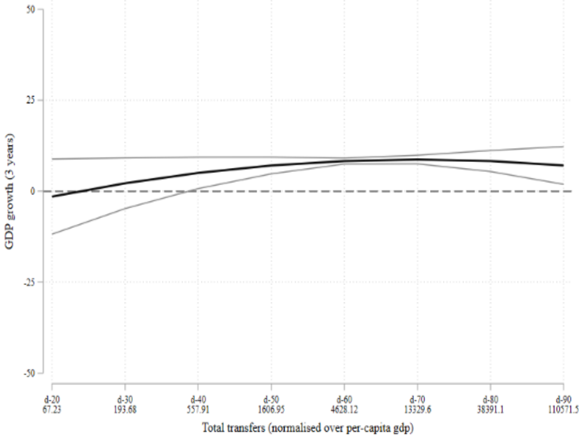

(d) Outcome: GDP growth rate (3 years)

Notes: The figures show the relationship between well-being growth rate over two years (panel a) or three years (panel c) or per-capita GDP growth rate over two years (panel b) or three years (panel d) and the total transfers' intensity. The expenditure variable is computed as a rolling sum in the two or three preceding years. The estimated model is a polynomial regression of order 2. The thick lines represent the point estimates and the thin lines represent the respective 95% confidence intervals. The dashed line identifies y=zero. Intensity variable is rescaled over the beginning-the-period per-capita GDP in PPS and then min-max normalised into the (0-100) interval. X-axis labels report the normalised expenditure dose and the respective transfers' ratio computed considering the rolling sums of the transfers.

Figure A.3 Well-being and GDP response to total transfers (ESF+ERDF) over two and three years

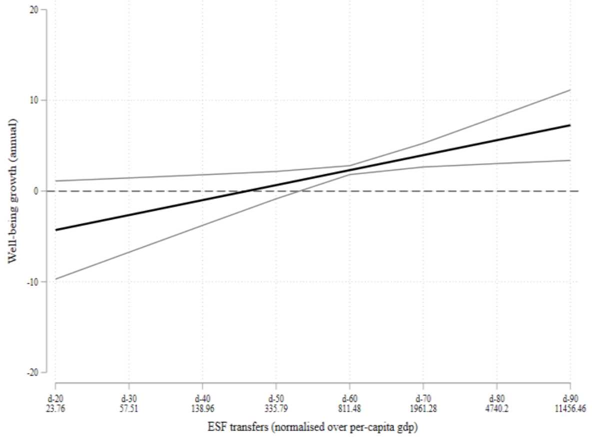

(a) Outcome: annual well-being growth rate

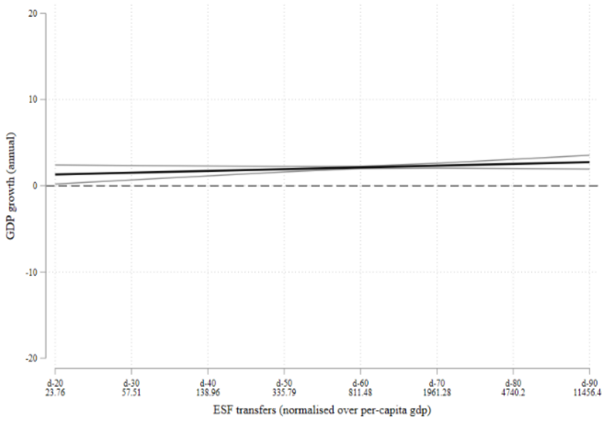

(b) Outcome: annual GDP growth rate

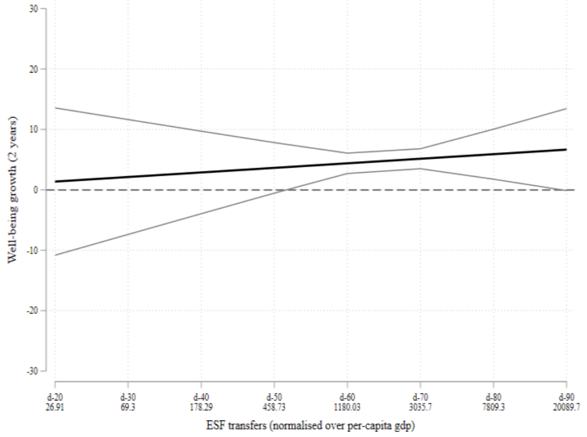

(c) Outcome: well-being growth rate (2 years)

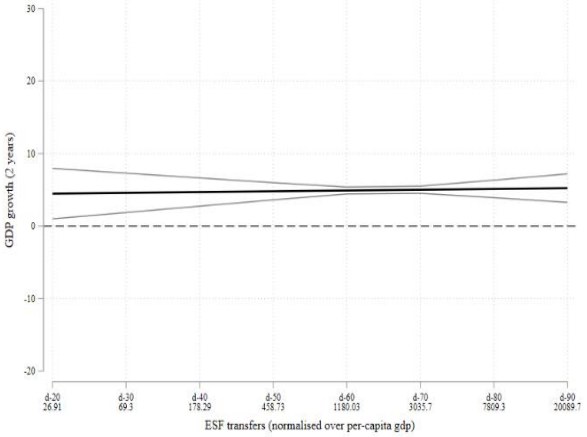

(d) Outcome: GDP growth rate (2 years)

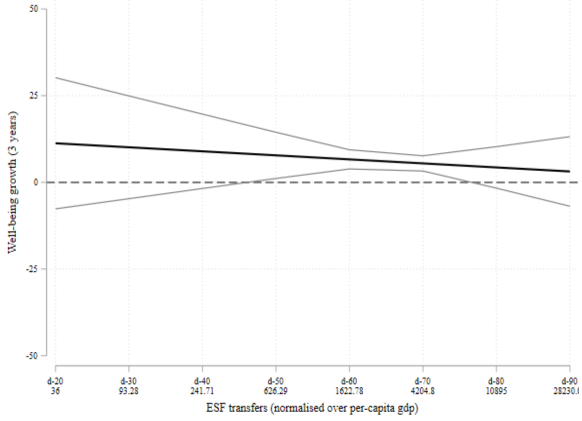

(e) Outcome: well-being growth rate (3 years)

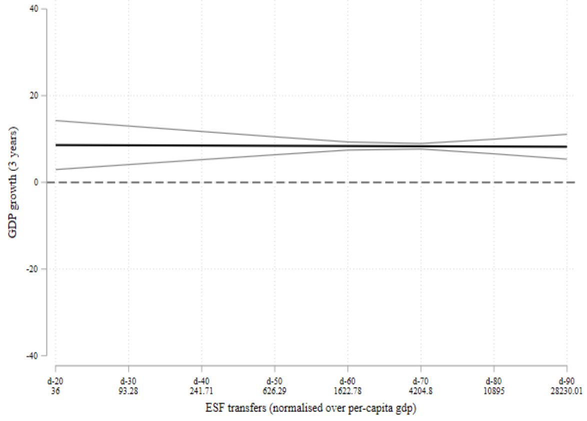

(f) Outcome: GDP growth rate (3 years)

Notes: The figures show the relationship between the well-being growth rate (panel a, c, e) or per-capita GDP (panel b, d, f) and the ESF transfers' intensity. ERDF is fixed at its mean value. The outcome variable is the annual growth rate (panel a, b) or the growth over two (panel c, d) or three years (panel e, f). The expenditure variable is one-period lagged (panel a, b) or computed as a rolling sum in the two (panel c, d) or three preceding years (panel e, f). The estimated model is a polynomial regression of order 2 that includes the interaction between the two transfers. The thick lines represent the point estimates and the thin lines represent the respective 95% confidence intervals. The dashed line identifies y=zero. Intensity variable is rescaled over the beginning-the-period per-capita GDP in PPS and then min-max normalised into the (0-100) interval. X-axis labels report the normalised expenditure dose and the respective transfers' ratio. In panel c-f, x-axis labels report the normalised expenditure dose and the respective transfers' ratio computed considering the rolling sums of the transfers.

Figure A.4 Well-being and GDP response ESF transfers

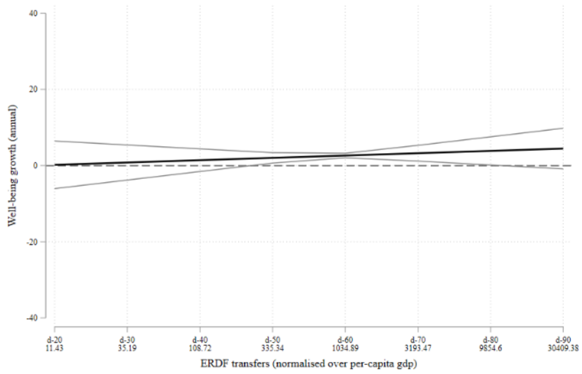

(a) Outcome: annual well-being growth rate

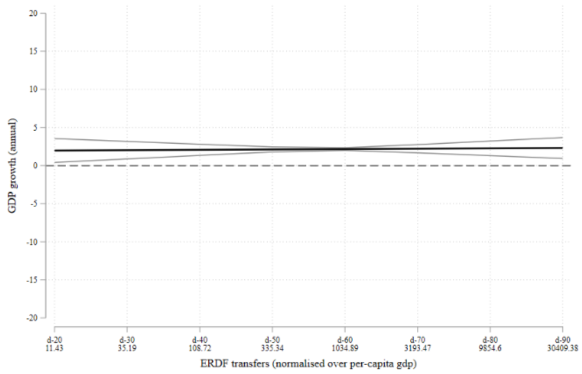

(b) Outcome: annual GDP growth rate

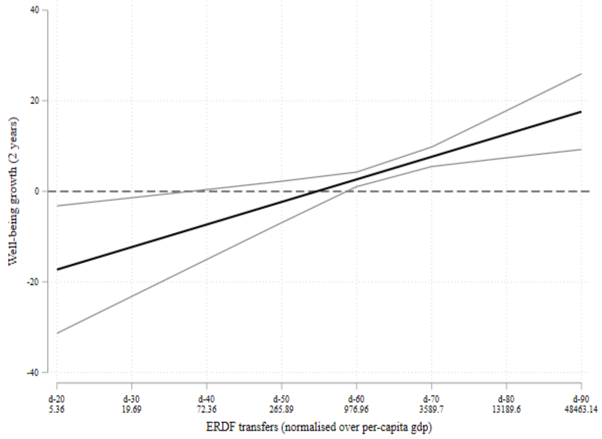

(c) Outcome: well-being growth rate (2 years)

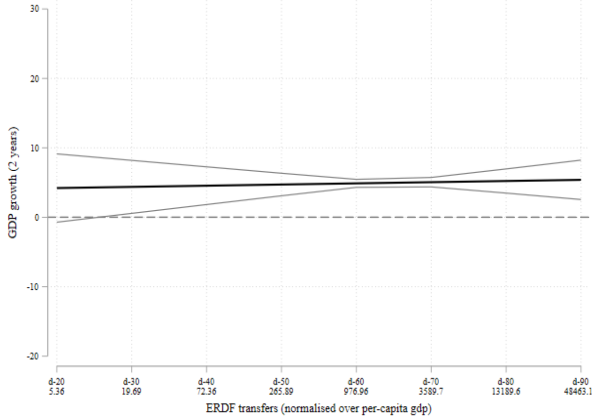

(d) Outcome: GDP growth rate (2 years)

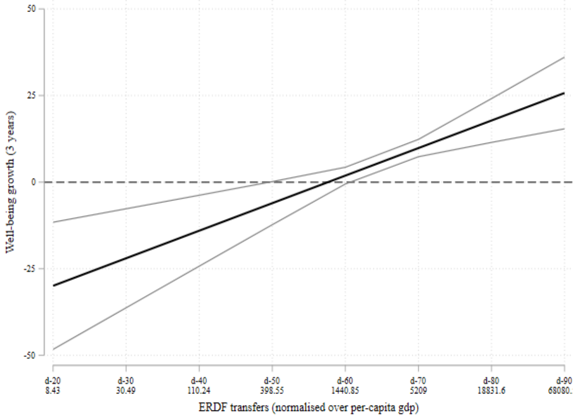

(e) Outcome: well-being growth rate (3 years)

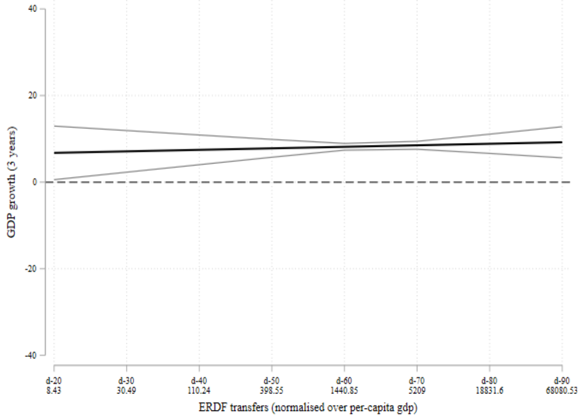

(f) Outcome: GDP growth rate (3 years)

Notes: The figures show the relationship between well-being growth rate (panel a, c, e) or per-capita GDP (panel b, d, f) and the ERDF transfers' intensity. ESF is fixed at its mean value. The outcome variable is the annual growth rate (panel a, b) or the growth over two (panel c, d) or three years (panel e, f). The expenditure variable is one-period lagged (panel a, b) or computed as a rolling sum in the two (panel c, d) or three preceding years (panel e, f). The estimated model is a polynomial regression of order 2 that includes the interaction between the two transfers. The thick lines represent the point estimates and the thin lines represent the respective 95% confidence intervals. The dashed line identifies y=zero. Intensity variable is rescaled over the beginning-the-period per-capita GDP in PPS and then min-max normalised into the (0-100) interval. X-axis labels report the normalised expenditure dose and the respective transfers' ratio. In panel c-f, x-axis labels report the normalised expenditure dose and the respective transfers' ratio computed considering the rolling sums of the transfers.

Figure A.5 Well-being and GDP response ERDF transfers

Tables

Table A.1 List of regions dropped by the sample in each specification

**Model**	**Dropped NUTS**
Parametric estimation, (Tables 2, A2, A3 cols. 5, 6 and Table A6 cols. 5, 6, 7, 8)	AT12, AT13, AT21, AT22, AT31, AT32, AT33, AT34, BE10, BE21, BE22, BE23, BE24, BE25, BE31, BE32, BE33, BE34, BE35, DE11, DE12, DE13, DE14, DE21, DE22, DE23, DE24, DE25, DE26, DE27, DE30, DE40, DE50, DE60, DE72, DE73, DE80, DE91, DE92, DE93, DE94, DEA1, DEA2, DEA3, DEA4, DEA5, DEB1, DEB2, DEB3, DEC0, DED2, DED4, DED5, DEE0, DEF0, DEG0, DK01, DK02, DK03, DK04, DK05, EL30, EL41, EL42, EL43, EL51, EL52, EL53, EL54, EL61, EL62, EL63, EL64, EL65, ES11, ES12, ES13, ES21, ES22, ES23, ES24, ES30, ES41, ES42, ES43, ES51, ES52, ES53, ES61, ES62, ES70, FI1B, FI1C, FI1D, FR10, FR21, FR22, FR23, FR24, FR25, FR26, FR30, FR41, FR42, FR43, FR51, FR52, FR53, FR61, FR62, FR63, FR71, FR72, FR81, FR82, IE01, IE02, ITC1, ITC2, ITC3, ITC4, ITF1, ITF2, ITF3, ITF4, ITF5, ITF6, ITG1, ITG2, ITH1, ITH2, ITH3, ITH4, ITH5, ITI1, ITI2, ITI3, ITI4, LU00, NL11, NL12, NL13, NL21, NL22, NL23, NL31, NL32, NL33, NL34, NL41, NL42, PT11, PT15, PT16, PT17, PT18, SE11, SE12, SE21, SE22, SE23, UKC1, UKC2, UKD1, UKD3, UKD4, UKD6, UKD7, UKE1, UKE2, UKE3, UKE4, UKF1, UKF2, UKF3, UKG1, UKG2, UKG3, UKH1, UKH2, UKH3, UKJ1, UKJ2, UKJ3, UKJ4, UKK1, UKK2, UKK3, UKK4, UKL1, UKL2, UKM2, UKM3, UKM5, UKM6, UKN0
Parametric estimation, (Tables 2, A2, A3, A6 cols. 1-4)	AT12, AT13, AT21, AT22, AT31, AT32, AT33, AT34, BE10, BE21, BE22, BE23, BE24, BE25, BE31, BE32, BE33, BE34, BE35, DE11, DE12, DE13, DE14, DE21, DE22, DE23, DE24, DE25, DE26, DE27, DE30, DE40, DE50, DE60, DE71, DE72, DE73, DE80, DE91, DE92, DE93, DE94, DEA1, DEA2, DEA3, DEA4, DEA5, DEB1, DEB2, DEB3, DEC0, DED2, DED4, DED5, DEE0, DEF0, DEG0, DK01, DK02, DK03, DK04, DK05, EL30, EL41, EL42, EL43, EL51, EL52, EL53, EL54, EL61, EL62, EL63, EL64, EL65, ES11, ES12, ES13, ES21, ES22, ES23, ES24, ES30, ES41, ES42, ES43, ES51, ES52, ES53, ES61, ES62, ES70, FI19, FI1B, FI1C, FI1D, FR10, FR21, FR22, FR23, FR24, FR25, FR26, FR30, FR41, FR42, FR43, FR51, FR52, FR53, FR61, FR62, FR63, FR71, FR72, FR81, FR82, IE01, IE02, ITC1, ITC2, ITC3, ITC4, ITF1, ITF2, ITF3, ITF4, ITF5, ITF6, ITG1, ITG2, ITH1, ITH2, ITH3, ITH4, ITH5, ITI1, ITI2, ITI3, ITI4, LU00, NL11, NL12, NL13, NL21, NL22, NL23, NL31, NL32, NL33, NL34, NL41, NL42, PT11, PT15, PT16, PT17, PT18, SE11, SE12, SE21, SE22, SE23, UKC1, UKC2, UKD1, UKD3, UKD4, UKD6, UKD7, UKE1, UKE2, UKE3, UKE4, UKF1, UKF2, UKF3, UKG1, UKG2, UKG3, UKH1, UKH2, UKH3, UKJ1, UKJ2, UKJ3, UKJ4, UKK1, UKK2, UKK3, UKK4, UKL1, UKL2, UKM2, UKM3, UKM5, UKM6, UKN0
DRF to total transfers, (Table A.5, cols. 1, 4)	AT12, AT13, AT21, AT22, AT31, AT32, AT33, AT34, BE10, BE21, BE22, BE23, BE24, BE25, BE31, BE32, BE33, BE34, BE35, DE11, DE12, DE13, DE14, DE21, DE22, DE23, DE24, DE25, DE26, DE27, DE30, DE40, DE50, DE60, DE71, DE72, DE73, DE80, DE91, DE92, DE93, DE94, DEA1, DEA2, DEA3, DEA4, DEA5, DEB1, DEB2, DEB3, DEC0, DED2, DED4, DED5, DEE0, DEF0, DEG0, DK01, DK02, DK03, DK04, DK05, EL30, EL41, EL42, EL43, EL51, EL52, EL53, EL54, EL61, EL62, EL63, EL64, EL65, ES11, ES12, ES13, ES21, ES22, ES23, ES24, ES30, ES41, ES42, ES43, ES51, ES52, ES53, ES61, ES62, ES70, FI19, FI1B, FI1C, FI1D, FR10, FR21, FR22, FR23, FR24, FR25, FR26, FR30, FR41, FR42, FR43, FR51, FR52, FR53, FR61, FR62, FR63, FR71, FR72, FR81, FR82, IE01, IE02, ITC1, ITC2, ITC3, ITC4, ITF1, ITF2, ITF3, ITF4, ITF5, ITF6, ITG1, ITG2, ITH1, ITH2, ITH3, ITH4, ITH5, ITI1, ITI2, ITI3, ITI4, LU00, NL11, NL12, NL13, NL21, NL22, NL23, NL31, NL32, NL33, NL34, NL41, NL42, PT11, PT15, PT16, PT17, PT18, SE11, SE12, SE21, SE22, SE23, UKC1, UKC2, UKD1, UKD3, UKD4, UKD6, UKD7, UKE1, UKE2, UKE3, UKE4, UKF1, UKF2, UKF3, UKG1, UKG2, UKG3, UKH1, UKH2, UKH3, UKJ1, UKJ2, UKJ3, UKJ4, UKK1, UKK2, UKK3, UKK4, UKL1, UKL2, UKM2, UKM3, UKM5, UKM6, UKN0
DRF to ESF, (Table A.5, cols. 2, 5)	AT12, AT13, AT21, AT22, AT31, AT32, AT33, AT34, BE10, BE21, BE22, BE23, BE24, BE25, BE31, BE32, BE33, BE34, BE35, DE11, DE12, DE13, DE14, DE21, DE22, DE23, DE24, DE25, DE26, DE27, DE30, DE40, DE50, DE60, DE72, DE73, DE80, DE91, DE92, DE93, DE94, DEA1, DEA2, DEA3, DEA4, DEA5, DEB1, DEB2, DEB3, DEC0, DED2, DED4, DED5, DEE0, DEF0, DEG0, DK01, DK02, DK03, DK04, DK05, EL30, EL41, EL42, EL43, EL51, EL52, EL53, EL54, EL61, EL62, EL63, EL64, EL65, ES11, ES12, ES13, ES21, ES22, ES23, ES24, ES30, ES41, ES42, ES43, ES51, ES52, ES53, ES61, ES62, ES70, FI19, FI1B, FI1C, FI1D, FR10, FR21, FR22, FR23, FR24, FR25, FR26, FR30, FR41, FR42, FR43, FR51, FR52, FR53, FR61, FR62, FR63, FR71, FR72, FR81, FR82, IE01, IE02, ITC1, ITC2, ITC3, ITC4, ITF1, ITF2, ITF3, ITF4, ITF5, ITF6, ITG1, ITG2, ITH1, ITH2, ITH3, ITH4, ITH5, ITI1, ITI2, ITI3, ITI4, LU00, NL11, NL12, NL13, NL21, NL22, NL23, NL31, NL32, NL33, NL34, NL41, NL42, PT11, PT15, PT16, PT17, PT18, SE11, SE12, SE21, SE22, SE23, SE32, UKC1, UKC2, UKD1, UKD3, UKD4, UKD6, UKD7, UKE1, UKE2, UKE3, UKE4, UKF1, UKF2, UKF3, UKG1, UKG2, UKG3, UKH1, UKH2, UKH3, UKJ1, UKJ2, UKJ3, UKJ4, UKK1, UKK2, UKK3, UKK4, UKL1, UKL2, UKM2, UKM3, UKM5, UKM6, UKN0
DRF to ERDF, (Table A.5, cols. 3, 6)	AT12, AT13, AT21, AT22, AT31, AT32, AT33, AT34, BE10, BE21, BE22, BE23, BE24, BE25, BE31, BE32, BE33, BE34, BE35, DE11, DE12, DE13, DE14, DE21, DE22, DE23, DE24, DE25, DE26, DE27, DE30, DE40, DE50, DE60, DE71, DE72, DE73, DE80, DE91, DE92, DE93, DE94, DEA1, DEA2, DEA3, DEA4, DEA5, DEB1, DEB2, DEB3, DEC0, DED2, DED4, DED5, DEE0, DEF0, DEG0, DK01, DK02, DK03, DK04, DK05, EL30, EL41, EL42, EL43, EL51, EL52, EL53, EL54, EL61, EL62, EL63, EL64, EL65, ES11, ES12, ES13, ES21, ES22, ES23, ES24, ES30, ES41, ES42, ES43, ES51, ES52, ES53, ES61, ES62, ES70, FI1B, FI1C, FI1D, FR10, FR21, FR22, FR23, FR24, FR25, FR26, FR30, FR41, FR42, FR43, FR51, FR52, FR53, FR61, FR62, FR63, FR71, FR72, FR81, FR82, IE01, IE02, ITC1, ITC2, ITC3, ITC4, ITF1, ITF2, ITF3, ITF4, ITF5, ITF6, ITG1, ITG2, ITH1, ITH2, ITH3, ITH4, ITH5, ITI1, ITI2, ITI3, ITI4, LU00, NL11, NL12, NL13, NL21, NL22, NL23, NL31, NL32, NL33, NL34, NL41, NL42, PT11, PT15, PT16, PT17, PT18, SE11, SE12, SE21, SE22, SE23, SE31, SE33, UKC1, UKC2, UKD1, UKD3, UKD4, UKD6, UKD7, UKE1, UKE2, UKE3, UKE4, UKF1, UKF2, UKF3, UKG1, UKG2, UKG3, UKH1, UKH2, UKH3, UKJ1, UKJ2, UKJ3, UKJ4, UKK1, UKK2, UKK3, UKK4, UKL1, UKL2, UKM2, UKM3, UKM5, UKM6, UKN0

Notes: This table reports the code of NUTS 2 regions dropped from the estimation sample for the baseline model in the respective table. The observations listed above are missing in a specific year and not necessarily throughout the whole period of analysis. A more detailed list is available from authors upon request.

Table A.2 Likelihood-ratio tests: Comparison between the unrestricted model and the restricted models

Outcome: Annual g(WB)

(a) Total

LRTEST [3,4]	LR test	T-Statistics	p-value	Restrictions
Unrestricted	-3478.14			
Covariates	-3701.64	447.00	0.000	9
GPS terms	-4176.81	1397.33	0.000	3
N	1656			

(c) ESF

LRTEST [3,4]	LR test	T-Statistics	p-value	Restrictions
Unrestricted	-3975.73			
Covariates	-4286.32	621.17	0.000	10
GPS terms	-4103.53	255.60	0.000	3
N	1596			

(e) ERDF

LRTEST [3,4]	LR test	T-Statistics	p-value	Restrictions
Unrestricted	-3000.61			
Covariates	-3281.86	562.49	0.000	10
GPS terms	-3472.39	943.56	0.000	3
N	1588			

Outcome: Annual g(GDP)

(b) Total

LRTEST [3,4]	LR test	T-Statistics	p-value	Restrictions
Unrestricted	-3143.15			
Covariates	-3225.12	163.95	0.000	8
GPS terms	-3865.17	1444.04	0.000	3
N	1545			

(d) ESF

LRTEST [3,4]	LR test	T-Statistics	p-value	Restrictions
Unrestricted	-4214.94			
Covariates	-4545.54	661.20	0.000	9
GPS terms	-4359.02	288.16	0.000	3
N	1698			

(f) ERDF

LRTEST [3,4]	LR test	T-Statistics	p-value	Restrictions
Unrestricted	-2830.4548			
Covariates	-3085.9717	511.03	0.000	9
GPS terms	-3490.62	1320.33	0.000	3
N	1607			

Notes: The table shows a likelihood ratio test to assess the balancing property of the propensity score by comparing restricted and unrestricted models. The outcome variable is the annual well-being growth rate (panels a, c, e) or the annual GDP growth rate (panels b, d, f).

Table A.3 Inverse-Weighted Kernel estimates of the Dose-Response Function

	(1) Annual g(WB)	(2) Annual g(GDP)	(3) Annual g(WB)	(4) Annual g(GDP)	(5) Annual g(WB)	(6) Annual g(GDP)
Intensity	-0.704	1.710***	-0.719	1.685***	-0.739	1.024***
	(0.473)	(0.229)	(0.539)	(0.208)	(0.539)	(0.242)
DRF-Intensity^2	0.219**	-0.265***	0.124	-0.244***	0.353***	-0.202***
	(0.096)	(0.049)	(0.105)	(0.014)	(0.134)	(0.062)
DRF-Intensity^3	-0.015**	0.015***	-0.006	0.014***	-0.031***	0.014***
	(0.006)	(0.003)	(0.007)	(0.003)	(0.010)	(0.004)
DRF-Intensity^4	0.0003**	-0.0003***	0.000	-1.018***	0.001***	-0.0003***
	(0.0001)	(0.000)	(0.000)	(0.289)	(0.000)	(0.000)
Treatment	Total	Total	ESF	ESF	ERDF	ERDF
Bandwidth weighting	1.359	0.883	1.8219	0.972	1.071	0.342
R-squared	0.008	0.071	0.1079	0.092	0.013	0.020
Number of Nuts2	1,492	1,366	1,426	1,493	1,424	1,411

Notes: IW kernel estimates of the dose-response function to Cohesion policy transfers, obtained applying the Stata routine by Bia et al. (2014b). The outcome variable is annual well-being growth (Column 1, 3, 5) or annual GDP growth (Column 2, 4, 6). Treatment is based on total transfers (Column 1, 2), ESF (Column 3, 4) or ERDF (Column 5, 6). In Columns 3 - 6, a variable indicating the quartile of the distribution of the other fund to which a region belongs is included among the matching pre-treatment covariates. Bootstrapped standard errors with 500 replications are reported in parenthesis. ***, **, and * indicate significance at the 1%, 5%, and 10% level, respectively.

Table A.4 Parametric estimation of the response function in terms of regional well-being and GDP growth rate (2 years) to Cohesion Policy transfers

	(1)	(2)	(3)	(4)	(5)	(6)
	g(WB) (2-years)	g(WB) (2-years)	g(GDP) (2-years)	g(GDP) (2-years)	g(WB) (2-years)	g(GDP) (2-years)
Total intensity	0.4428**	1.1606**	0.0192	0.3575**		
	(0.172)	(0.506)	(0.058)	(0.169)		
		-0.0061		-0.0029**		
Total intensity squared		(0.004)		(0.001)		
ESF intensity					0.6033**	0.1731*
					(0.291)	(0.095)
ERDF intensity					1.0311***	0.1810*
					(0.324)	(0.102)
ESF × ERDF					-0.0082**	-0.0025*
					(0.004)	(0.001)
Observations	1,471	1,471	1,444	1,444	1,475	1,448
R-squared	0.23520	0.23670	0.80105	0.80229	0.25296	0.80798
Number of Nuts2	193	193	193	193	194	194

Notes This table presents the effect on the intensive margins of total (ESF+ERDF) transfers intensity on the annual well-being growth rate (columns 1-2) and per-capita GDP growth rate (columns 3-4). Columns 5 - 6 look at the effect by single funding stream. Both a linear (columns 1, 3, 5, 6) and a quadratic function (columns 2, 4) of the intensity are proposed. The intensity variable is rescaled over the beginning-the-period per-capita GDP in PPS and then min-max normalised into the (0-100) interval. Across columns, all models include regional and year-fixed effects and linear country trends. Expenditure variables are computed as two-year rolling sum. Standard errors, clustered at the regional level, are shown in parentheses. ***, **, and * indicate significance at the 1%, 5%, and 10% level, respectively.

Table A.5 Parametric estimation of the response function in terms of regional well-being and GDP growth rate (3 years) to Cohesion Policy transfers

	(1)	(2)	(3)	(4)	(5)	(6)
	g(WB) (3-years)	g(WB) (3-years)	g(GDP) (3-years)	g(GDP) (3-years)	g(WB) (3-years)	g(GDP) (3-years)
Total intensity	0.4134	2.0895**	0.0832	0.5690**		
	(0.278)	(0.908)	(0.089)	(0.286)		
		-0.0142**		-0.0041*		
Total intensity squared		(0.007)		(0.002)		
ESF intensity					0.7751*	0.2684*
					(0.461)	(0.155)
ERDF intensity					1.7021***	0.3142*
					(0.457)	(0.178)
ESF × ERDF					-0.0137**	-0.0042*
					(0.006)	(0.002)
Observations	1,285	1,285	1,258	1,258	1,306	1,279
R-squared	0.22241	0.22746	0.80961	0.81101	0.24191	0.81413
Number of Nuts2	193	193	193	193	194	194

Notes: This table presents the effect on the intensive margins of total (ESF+ERDF) transfers intensity on the annual well-being growth rate (columns 1-2) and per-capita GDP growth rate (columns 3-4). Columns 5 - 6 look at the effect by single funding stream. Both a linear (columns 1, 3, 5, 6) and a quadratic function (columns 2, 4) of the intensity is proposed. Intensity variable is rescaled over the beginning-the-period per-capita GDP in PPS and then min-max normalised into the (0-100) interval. Across columns all models include regional and year fixed effects and linear country trends. Expenditure variables are computed as three-year rolling sum. Standard errors, clustered at regional level, are shown in parentheses. ***, **, and * indicate significance at the 1%, 5%, and 10% level, respectively.

Table A.6 Parametric estimation of the response function in terms of regional well-being and GDP growth rate to Cohesion Policy transfers. Heterogeneity on Objective 1 regions.

	(1) Annual g(WB)	(2) Annual g(WB)	(3) Annual g(GDP)	(4) Annual g(GDP)	(5) Annual g(WB)	(6) Annual g(WB)	(7) Annual g(GDP)	(8) Annual g(GDP)
Intensity	0.3778***	0.6086*	0.0244	0.2743***	0.2033**	0.0279	0.0042	0.0050
	(0.103)	(0.368)	(0.024)	(0.095)	(0.082)	(0.059)	(0.016)	(0.022)
Intensity × OBJ1	-0.1075	-0.1671	0.0201	0.0738	-0.1086	0.1892**	0.0113	0.0249
	(0.105)	(0.619)	(0.032)	(0.170)	(0.089)	(0.083)	(0.023)	(0.038)
Intensity squared		-0.0022		-0.0024***				
		(0.003)		(0.001)				
Intensity sq × OBJ1		0.0009		0.0000				
		(0.005)		(0.001)				
Linear combination	0.270**	0.44	0.0445	0.346**	0.0947	0.217**	0.0155	0.0299
p-value	0.0440	0.380	0.164	0.0162	0.208	0.0345	0.465	0.452
Treatment	Total transfers	Total transfers	Total transfers	Total transfers	ESF	ERDF	ESF	ERDF
Observations	1,622	1,622	1,597	1,597	1,677	1,648	1,644	1,623
R-squared	0.17077	0.17102	0.73643	0.73897	0.17422	0.15121	0.74329	0.7029
Number of Nuts2	191	191	191	191	200	193	200	193

Notes: This table presents the effect on the intensive margins of total (ESF+ERDF) transfers intensity on the annual well-being growth rate (columns 1-2) and per-capita GDP growth rate (columns 3-4). Columns 5 - 8 look at the effect by single funding stream. Both a linear (columns 1, 3, 5, 6, 7, 8) and a quadratic function (columns 2, 4) of the intensity is proposed. Intensity variable is rescaled over the beginning-the-period per-capita GDP in PPS and then min-max normalised into the (0-100) interval. OBJ1 is a dummy variable identifying those regions eligible for the Objective 1 status in the programming period 2000-2006. Across columns all models include regional and year fixed effects and linear country trends. Expenditure variables are one-year lagged. Standard errors, clustered at regional level, are shown in parentheses. ***, **, and * indicate significance at the 1%, 5%, and 10% level, respectively.
